# Oxidation of Iodine to Dihaloiodate(I) Salts of Amines With Hydrogen Peroxides and Their Crystal Structures

**DOI:** 10.3389/fchem.2022.912383

**Published:** 2022-05-05

**Authors:** Griša Grigorij Prinčič, Nik Maselj, Evgeny Goreshnik, Jernej Iskra

**Affiliations:** ^1^ Faculty of Chemistry and Chemical Technology, University of Ljubljana, Ljubljana, Slovenia; ^2^ Department of Inorganic Chemistry and Technology, Jožef Stefan Institute, Ljubljana, Slovenia

**Keywords:** Hypervalent iodine compounds, dihaloiodates, halogenation, hydrogen peroxide, crystal structure, amine base, halogen bonds

## Abstract

Herein we report a general preparation of dihaloiodate salts of heterocyclic amines (tertiary and quaternary) with sterically accessible and hindered nitrogen atom. A number of such compounds were prepared from preformed HICl_2_ or HIBr_2_ formed *in situ* by the reaction of corresponding hydrogen halide, iodine and H_2_O_2_. The salts of 1,4-diazabicyclo[2.2.2]octane (DABCO) and its methylated derivatives, 1,3,5,7-tetraazaadamantane (HMTA), diazabicycloundecene (DBU) and 2,4,6-tri-*tert*-butylpyridine (TBP) were obtained in excellent yields and their structure was determined by NMR and Raman spectroscopy and single crystal X-ray diffraction. Non-hindered bases such as DABCO, HMTA and DBU formed IX_2_^−^ salts, which further decomposed to complexes with interhalogen compounds due to formation of N…X halogen bonds. The dihaloiodiate(I) salts of sterically hindered 2,4,6-tri-*tert*-butylpyridine were stable. Its dichlorobromate(I) salt was also prepared *via* a different synthetic method using *N*-chlorosuccinimide as oxidant.

## Introduction

Even today, one of the most important chemical reactions is the halogenation of organic compounds. Due to their properties, halogenated organic compounds are of great importance in synthetic and organic chemistry as well as in daily life (Gribble, 2003). Halogenation in industry and laboratory is mainly carried out with elemental halogens, which pose a significant hazard due to their reactivity, toxicity, and corrosiveness. Therefore, the development of new, greener and more sustainable halogenation processes and reagents is necessary (Podgoršek et al., 2009; Segura-Quezada et al., 2020; Scheide et al., 2021; Varenikov et al., 2021; Torregrosa-Chinillach and Chinchilla, 2022).

Hypervalent iodine compounds have iodine in higher oxidation states (I, III, V). They have slowly replaced the use of heavy metals such as lead and mercury in combination with acids for iodination reactions (Zhdankin and Wirth, 2016). Since then, a significant amount of research has been done in this field (Zhdankin and Stang, 2008; Zhdankin and Protasiewicz, 2014; Zhdankin and Wirth, 2016) and hypervalent iodine compounds in the oxidation states (III) and (V) are most commonly used as oxidants (e.g., Dess-Martin periodinane). Iodine(I) compounds, on the other hand, are more suitable as electrophilic iodination reagents (Barluenga, 1999; Kovalreagents, 2002; Bedrač and Iskra, 2013; Day et al., 2021). Because of the weakly nucleophilic nature of halogen anions (Cl^−^, Br^−^ and I^−^), halogenation reactions usually proceed *via* an electrophilic mechanism involving a halogen atom with a slightly positive character. For this reason, interhalogen compounds are promising for halo-functionalization. Trihalides are compounds consisting of three identical or different halogen atoms [XYZ]^−^ (X, Y, Z = F, Cl, Br or I) (Haller and Riedel, 2014; Sonnenberg et al., 2020). Trihalides with two identical ligands bonded to a central halogen atom [XY_2_]^−^ are particularly interesting and useful as halogenating reagents (Van den Bossche et al., 2018).

Dichloroiodates(I) (DCI) have been mainly used for iodination of various organic molecules, while ionic liquids containing trihalide anions have been used as oxidative solvents for dissolution of metals (Luo et al., 2016; Van den Bossche et al., 2018). While [1-butyl-3-methylimidazolium]ICl_2_^−^ was prepared as a recyclable ionic liquid for the iodination of activated anilines and heteroaromatic amines (Deshmukh et al., 2015). DCIs such as potassium (K^+^ICl_2_^−^) and tetramethylammonium (Me_4_N^+^ICl_2_^−^) salts are used for the iodination of unsaturated compounds such as alkenes (Kajigaeshi et al., 1990) and aromatic compounds (Garden et al., 2001; Quintin and Lewin, 2004; Miners et al., 2013; Sereda et al., 2020), while 18-crown-6 supported DCIs were developed as soluble counterparts. Me_4_N^+^ICl_2_^−^ is reported to be a very reactive iodination reagent, capable of iodinating 4-nitrotoluene in 35 min at room temperature. However, this increased reactivity can only be achieved in the presence of superacids (Filimonov et al., 2008). Benzyltrimethylammonium DCI (PhCH_2_Me_3_N^+^ICl_2_^−^) was prepared and used for the iodination of aromatic amines (Kajigaeshi et al., 1988a), fenols (Kajigaeshi et al., 1987), aromatic ethers (Kajigaeshi et al., 1988b), acetanilides (Kajigaeshi et al., 1989) and even as a catalyst in the four-component synthesis of pyrroles (Pagadala and Kusampally, 2019). Recently, it was also used in the total synthesis of scutellarin (Liu et al., 2019), an enantioselective synthesis of islatravir (Patel et al., 2020) and secalonic acid (Qin and Porco, 2014). Polymer-bound DCI compounds have also been developed (Šket et al., 1989; Sumi Mitra and Sreekumar, 1997; Bahrami-Nasab and Pourali, 2014). DCI compounds function primarily as electrophilic iodination reagents, but dibromoiodates(I) (DBIs) react with alkenes to form brominated products. Dichloroiodic acid (HICl_2_) is a better iodinating reagent and has been shown to iodinate arenes, alkenes and alkynes. It can be easily prepared from iodine, concentrated aqueous HCl and 30% aqueous hydrogen peroxide (Bedrač et al., 2012).

Dihaloiodates are stable compounds and can be stored at ambient conditions. Tetraalkylammonium DCI decomposes only slightly in ethanol solutions, with stability decreasing with shorter alkyl chain length. Concentrated solutions of HICl_2_ in water and various organic solvents are also stable for days. Decomposition occurs at higher pH or in a biphasic system (e.g., dichloromethane-H_2_O) (Bedrač et al., 2012; Bedrač and Iskra, 2013).

A number of DCI and DBI crystal structures (and some difluoroiodates) are known. They consist of a linear or nearly linear IX_2_^−^ anion and a selected cation. In the anion, the average distance between the central iodine atom and the adjacent halogen atom is about 250 pm for DCI, 260 pm for DBI, and 200 pm in the case of difluoroiodates. Some examples of known dihaloiodates crystal structures are potassium DCI, its monohydrated form, potassium DBI monohydrate, and DCI and DBI salts with 2,2′-bipyridine (Soled and Carpenter, 1973a; Soled and Carpenter, 1973b; Soled and Carpenter, 1974). An example of a DCI salt with a crown ether is cesium (18-crown-6)dichloroiodate (El Essawi and Tebbe, 1998). The potassium analog is also known (Mbatia et al., 2011). Both have a similar structure in which the ICl_2_^−^ is sandwiched between two crown ether cations. The linear dihaloiodate anion is not always symmetrical. The ICl_2_^−^ structure in piperazinium bis (dichloroiodate) has I−Cl distances of 269 and 247 pm (Rømming et al., 1958).

**SCHEME 1:**
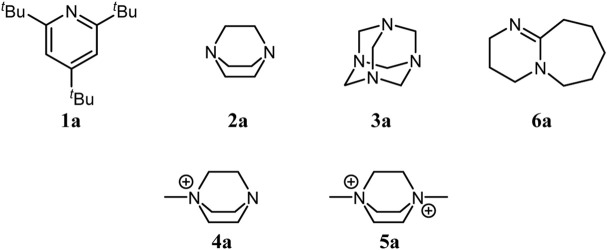
Amine bases used in the synthesis of ammonium dihaloiodates(I).

In view of these reports and the lack of data on properties and crystal structures of dihaloiodates(I), we have prepared several new dihaloiodate(I) (ICl_2_^−^, IBr_2_^−^) and dichlorobromate(I) (BrCl_2_^−^) salts with 2,4,6-tri-*tert*-butylpyridine (TBP) **1a**, 1,4-diazabicyclo[2.2.2]octane (DABCO **2a**) and its derivatives, and hexamethylenetetramine (HMTA **3a**) using elemental iodine and 30% aqueous hydrogen peroxide as oxidant (Scheme 1). We determined their crystal structures using single crystal X-ray diffraction and measured Raman spectra. Packing of dihaloiodate-anions in combination with diamine cations was analyzed. Interesting decomposition products with nitrogen-halogen bonds were also observed and characterized.

All DCI and DBI salts were prepared by an acid-base reaction between HIX_2_ (X = Cl or Br) and an amine base. Both HICl_2_ and HIBr_2_ were prepared in MeCN solution from elemental iodine, 30% H_2_O_2_ and concentrated aqueous HCl or HBr (Eq. 1) (Bedrač and Iskra, 2013).

$$\begin{array}{l} I_{2}+H_{2}O_{2}\mathrm{+HX}\to \mathrm{HI}X_{2}+H_{2}O\\ \;\;\;\;\;\;\;\;\;\;\;\;\;\;\;\;\;\;\;\;\;\;\;\;\;\;\;\;\;\;\;\;\;\;\;\;\;\;\;\;\;\mathrm{X=Cl,Br} \end{array}$$ (1)

The source of iodine atoms was elemental iodine in the case of the tertiary amines (HMTA, DABCO and 2,4,6-tri-*tert*-butylpyridine)and the iodide ion (Q^+^I^−^) already present in the salt in the case of the quaternary amines (Scheme 1).

## Dichloroiodates(I) (DCI)

First, we investigated the synthesis of dichloroiodates(I) from dichloroiodic acid (HICl_2_) and an amine base. We have chosen monobasic, sterically hindered base—2,4,6-tri-*tert*-butylpyridine **1a** as we anticipated that the bulky character of the *tert*-butyl groups would prevent the formation of hydrogen bonds in the crystal structure. Then we also used di- and tri-basic amines and dibasic starting compounds that should form stable salts—bases such as DABCO **2a**, HMTA **3a** and DBU **6a**. As a third group of bases we used quaternary amines—monomethylated **4a** and dimethylated **5a** derivatives of DABCO. HICl_2_was prepared by reaction between iodine, hydrochloric acid and hydrogen peroxide and the corresponding amine was added to form the ICl_2_^−^ salt. Iodine was dissolved in acetonitrile and 2 or 4 equivalents of concentrated aqueous HCl was added followed by 1 or 2 equivalents of 30% aq. H_2_O_2_. The solution was stirred at room temperature for 3 h to complete the formation of HICl_2_, which formed as a yellow solution. The amine base was added and the salt was formed immediately. The solvent was removed under reduced pressure and the salts were washed with cold ethanol to remove water and unreacted starting material. When quaternary amines from methylated DABCO **4a** and **5a** (Scheme 1) were used as starting substrates, the source of the iodine atom was the iodide ion already present in the salt. Yields of all isolated compounds were over 87% (Table 1). In addition to the DCI salts, we also prepared mixed salts with two different anions in one formula unit Q^2+^ICl_2_^−^Cl^−^ **2c** and **4c** by adding 1 more equivalent of HCl to the reaction mixture (Table 1). The presence of the dichloroiodate anion in the structure was first confirmed with Raman spectra with the peak characteristic of the dichloroiodate anion at 272 cm^−1^ and 143 cm^−1^ for ICl_2_^−^ and by X-ray structure determination.

**TABLE 1:** Reaction conditions for preparation of dichloroiodates(I).

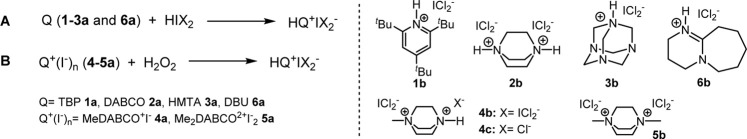					
**Entry**	**Base**	**Conditions**	**HCl (eq.)**	**Product**	**Yield (%)**
1	TBP **1a**	Method A^a^	2	Q^+^ICl_2_ **1b**	98
2	DABCO **2a**	Method A	4	Q^+^(ICl_2_)_2_ **2b**	92
3	DABCO **2a**	Method A	3	Q^2+^(ICl_2_)Cl^c^ **2c**	90
4	HMTA **3a**	Method A	2	Q^2+^ICl_2_ **3b**	95
5	MeDABCO **4a**	Method B^b^	4	Q^2+^(ICl_2_)_2_ **4b**	86
6	MeDABCO **4a**	Method B	3	Q^2+^ICl_2_Cl^c^ **4c**	87
7	Me_2_DABCO **5a**	Method B	4	Q^2+^(ICl_2_)_2_ **5b**	96
8	DBU **6a**	Method A	2	Q^+^ICl_2_ **6b**	90

a **Method A**: Iodine (127 mg, 0.5 mmol), 37% HCl (4 mmol), 30% H_2_O_2_ (1 mmol, 113 mg), MeCN (5 ml). Corresponding base **1a**, **2a**, **3a** or **6a** (1 mmol) was added at 0 °C and stirred for additional 10 min.

b **Method B**: Quaternary amine iodide **4a** or **5a** (1 mmol), 37% HCl (4 mmol), 30% H_2_O_2_ (1 mmol, 113 mg), MeCN (5 ml).

c For preparation of dichloroiodates(I) chlorides **2c** and **4c**, an additional equivalent of 37% HCl, was added to the reaction.

## Dibromoiodates(I) (DBI)

A similar procedure was used for the preparation of dibromoiodate(I) salts. Instead of HCl, HBr was added to the reaction mixture. Due to the easy oxidation of HBr, HIBr_2_ formed faster than HICl_2_ and the consumption of iodine was completed after 10 min at room temperature. After the iodine was consumed, the amines (**1−6a**) were added (Table 2, Method A) and the reaction was stirred for additional 10 min. The solvent was then removed under reduced pressure and the salts were washed with cold ethanol to remove water and unreacted starting material. As with the dichloroiodates(I), the source of iodine in the case of the quaternary ammonium salts was the iodide ion already present in the molecule (Table 2, Method B). Yields of all compounds were over 87%.

**TABLE 2:** Reaction conditions for preparation of dibromoiodates(I).

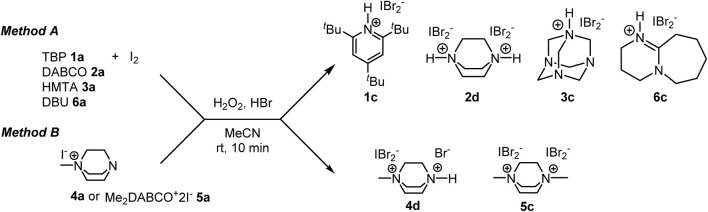					
**Entry**	**Base**	**Conditions**	**HBr (eq.)**	**Product**	**Yield (%)**
1	TBP **1a**	Method A^[a]^	2	Q^+^IBr_2_ **1c**	98
2	DABCO **2a**	Method A	4	Q^2+^(IBr_2_)_2_ **2d**	88
3	HMTA **3a**	Method A	2	Q^+^IBr_2_ **3c**	93
4	MeDABC**O 4a**	Method B^[b]^	4	Q^2+^IBr_2_Br **4d** ^[c]^	87
5	Me_2_DABCO **5a**	Method B	4	Q^2+^(IBr_2_)_2_ **5c**	92
6	DBU **6a**	Method B	2	Q^+^IBr_2_ **6c**	98

a **Method A**: Iodine (127 mg, 0.5 mmol), 48% HBr (4 mmol), 30% H_2_O_2_ (1 mmol, 113 mg), MeCN (5 ml). Corresponding base **1a**, **2a**, **3a** or **6a** (1 mmol) was added at 0 °C and stirred for additional 10 min.

b **Method B**: Quaternary amine iodide **4a** or **5a** (1 mmol), 48% HBr (4 mmol), 30% H_2_O_2_ (1 mmol, 113 mg), MeCN (5 ml)

c For preparation of dibromoiodates(I) bromide **4d**, an additional equivalent of 48% HBr was added to the reaction.

## Dichlorobromates(I) (DCB)

**SCHEME 2:**
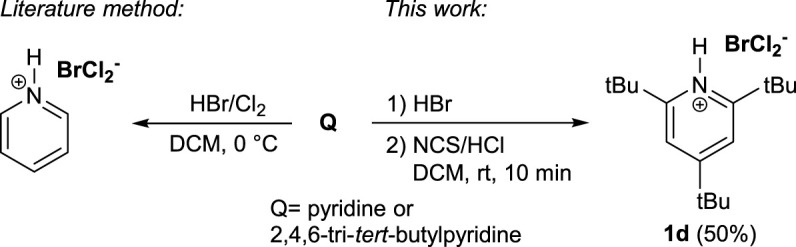
Method for preparation of dichlorobromates(I)

Last but not least, we investigated the preparation of dichlorobromate(I) salts (Q^+^BrCl_2_^−^). There are few data on these compounds in the literature. Most procedures for their preparation include reactions of bromides (or hydrobromides) with elemental chlorine in nonpolar solvents (Negoro and Ikeda, 1986; Muathen, 2002). To replace elemental chlorine, we instead used *N*-chlorosuccinimide (NCS) as an oxidant and amine base hydrochloride (Q^+^Cl^−^) (Scheme 2). **1a** was dissolved in DCM and 1 equivalent of 48% HBr was added. The reaction was stirred at room temperature for 20 min to complete the formation of the bromide salt. One equivalent of 37% HCl and 1.1 equivalents of NCS were then added. The reaction mixture immediately turned from colorless to bright yellow. The organic phase was extracted with water, dried with anhydrous Na_2_SO_4_ and evaporated under reduced pressure to give **1d** in 50% yield.

## Properties and Stability

All dichloroiodate(I) salts are bright yellow colored solids and, are stable in their solid form at room temperature. However, the compounds react slowly with wet solvents (DMSO, MeCN) and decompose within hours of dissolution. They are sparingly soluble in organic solvents such as MeCN, MeOH, and DMF and completely insoluble in chlorinated solvents (DCM, CHCl_3_). NMR spectra were recorded immediately after dissolution in DMSO-*d*_*6*_ and show broad peaks in ^1^H and ^13^C NMR spectra. Dichloroiodates(I) exhibit characteristic and strong signals in Raman spectra at about 140 and 260 cm^−1^. The structures were confirmed by X-ray spectroscopy and the structural features of the crystals are explained *vide infra* (Supporting Information).

Compared to the dichloroiodates(I), the dibromoiodates(I) were orange to dark red in color and were also stable compounds in solid form. They decomposed faster in solutions and were also more soluble in polar solvents such as MeCN and MeOH, forming orange-red solutions. Upon heating, they decompose and iodine is formed. NMR spectra were recorded immediately after dissolution in dry DMSO-*d*_*6*_ to prevent decomposition. A strong characteristic peak at about 160 cm^−1^ is visible in Raman spectra.

The dichlorobromates(I) salts (DCB) are the least stable compounds and decompose immediately after preparation. TBP salt **1d** is the only compound that is stable at room temperature and, like most interhalogen compounds, decomposes in wet solvents or upon heating. Unlike DCI and DBI, DCB **1d** is soluble in aprotic nonpolar solvents such as DCM and CHCl_3_. ^1^H and ^13^C spectra show much sharper signals and even the acidic proton is visible at 11.71 ppm. The Raman spectra show a strong signal at 171 cm^−1^. We also attempted the preparation of diethylDABCO bis[dichlorobromate(I)] by both methods. After the reaction at 0°C, bright yellow precipitate formed. However, when the suspension warmed to room temperature, it turned pale yellow, indicating decomposition. After crystallization of this pale-yellow product, the X-ray structure showed crystals of diethylDABCO^2+^Br_3_^−^Cl^−^. Due to the instability of BrCl_2_^−^, the compound decomposed into the more stable tribromide-chloride salt.

**SCHEME 3:**
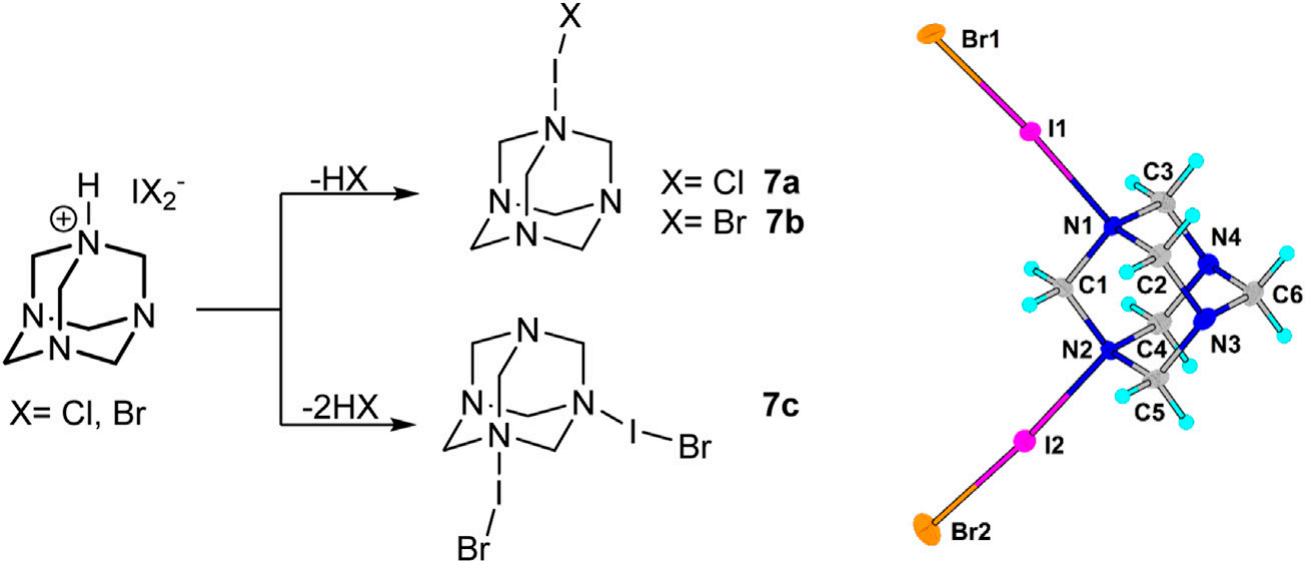
Formation of decomposition products **7** form dihaloiodates(I) **3** and the crystal structure of **7c.**

A similar change during the crystallization process was observed in the case of HMTA salts **3b** and **3c**. Instead of the formation of dihaloiodate(I) salts, interesting products **7a**, **7b**, and **7c** crystallized as dark orange crystals alongside the desired dihaloiodates(I) (Scheme 3). Dihaloiodate(I) ion decomposes with the release of hydrogen halide (HCl or HBr). The resulting iodine monochloride or monobromide forms a halogen bond with one or two nitrogen atoms in HMTA **3a**. The Raman spectra, however, did not show any discernable differences in resonances of halogen atoms between the compounds (Supplementary Picture S1).

## Crystal Structures

**FIGURE 1:**
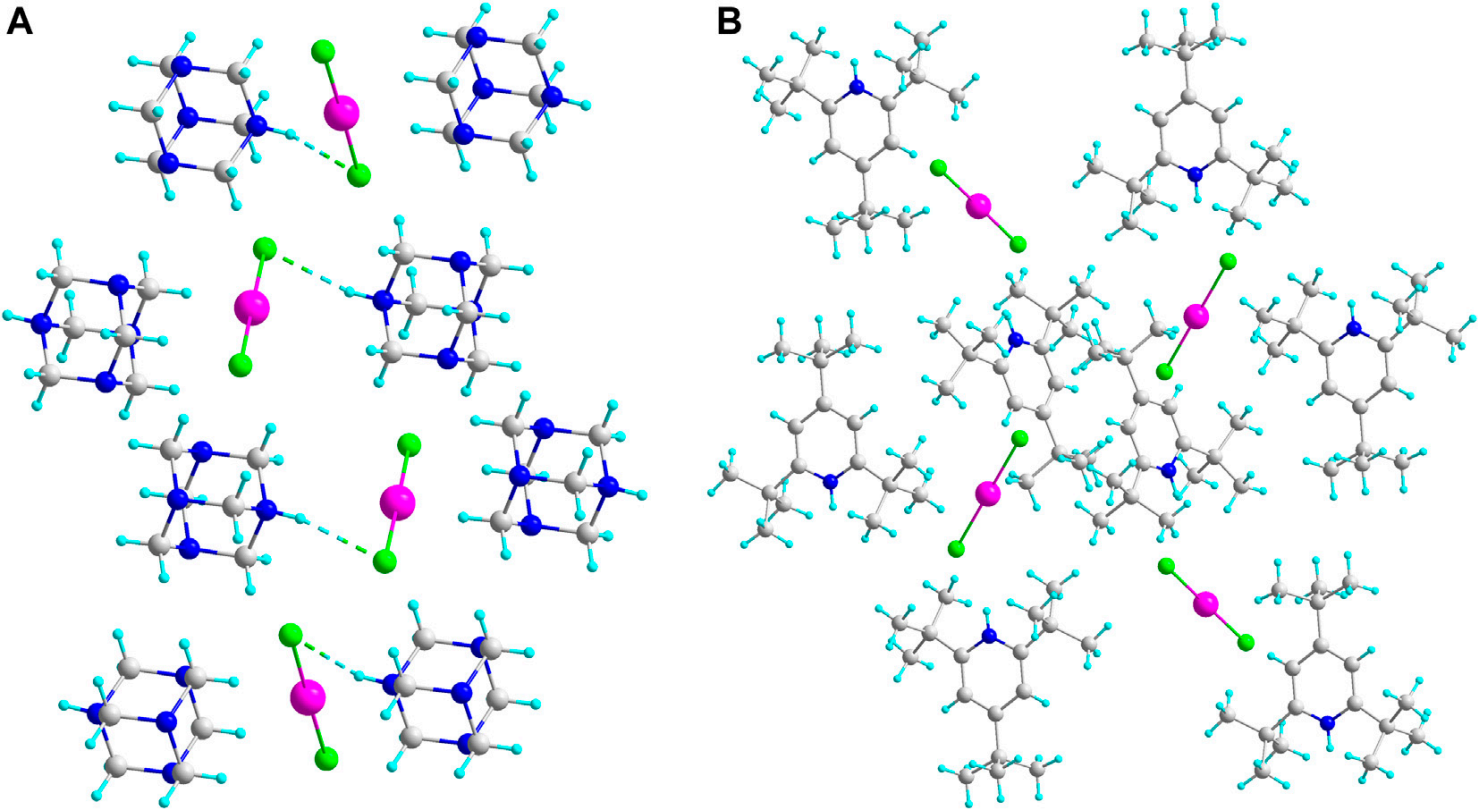
Comparison of crystal structures of HMTA DCI **3b (A)** and TBP DCI **1b (B)**. Hydrogen bonding network in **3b** is depicted with ----.

Geometric parameters of hydrogen bonds (*D*—H, H···*A* and *D*···*A* distances and *D*—H···*A* angles), and halogen bonds (*D*···*A* distances) are listed in Supplementary Table S4. The packing of the three-dimensional structure clearly depends on the nature of the cation. In the case of the bulky 2,4,6-tri-*tert*-butylpyridine scaffold, the cations and anions are packed without any noticeable cation-anion interactions (Figure 1). Evidently, the steric hindrances of the bulky organic moiety prevent the formation of hydrogen bonds between the protonated nitrogen atom and the inorganic anion. One should also mention that all these compounds with ICl_2_, IBr_2_ and BrCl_2_ anions are isotypical and crystallize in very similar unit cells (Supplementary Table S1), again indicating that the packing of the large organic part plays the main role in the formation of the three-dimensional structure. The N···Cl bond length in the ICl_2_^−^ salt of *H*_*2*_ *DABCO* ^*2+*^ is rather short because it involves isolated Cl^−^ anion. The N···Br distances in the IBr_2_^−^ analogue vary in rather wide range.

**FIGURE 2:**
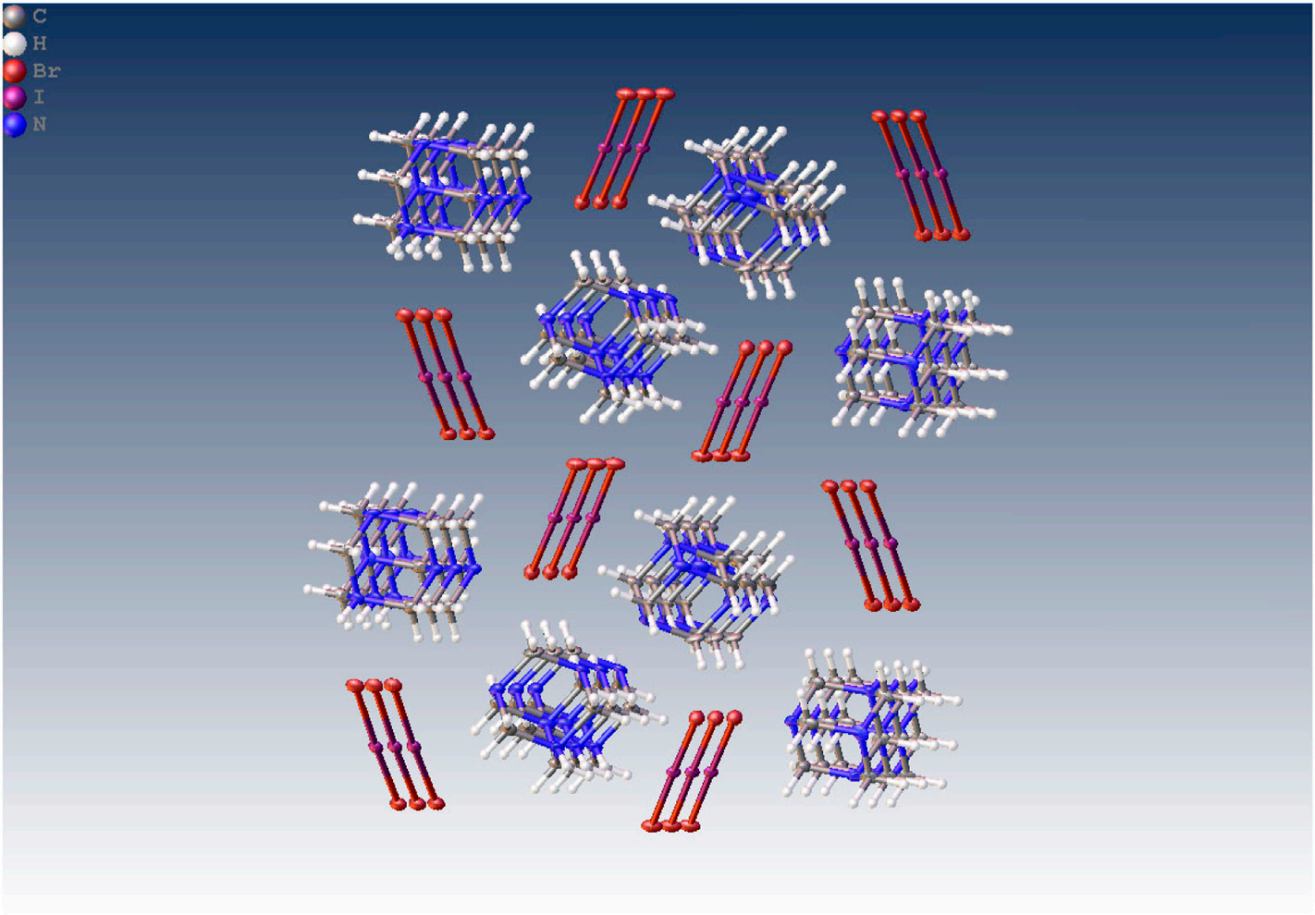
Crystal structure of **3c** with halogen bonding.

In contrast, in the case of monoprotonated hexamethylenetetramine, the spatially accessible hydrogen atoms form weak hydrogen bonds with anionic moiety (Figure 2). The N...Br distances in HMTA DBI **3c** are 3.385–3.408 Å. In HMTA DCI **3b**, the Cl...N distance is 3.267 Å. These two salts are not isotypical and even crystallize in different crystal systems. In both structures, the two nearest linear anions are mutually tilted, but the corresponding torsion angles (defined with respect to the terminal atoms of the two nearest anions) vary markedly: 92^o^ in the ICl_2_ salt and 43.8^o^ in the IBr_2_ derivative.

Interesting peculiarities are shown by the MeDABCO derivatives. In both **4c** and **4d**, the anions are located far from the organic cationic moiety and form weak C—H...Cl(Br) bonds. In the chloride derivative **4c**, the HCl molecule forms a Cl—H...N bond to the nitrogen center of the MeDABCO moiety (N...Cl 2.952 Å), while in the bromine salt **4d**, the hydrogen bromide molecule forms a weak Br—H...Br bond with the anion (Br...Br 4.078 Å).

The clearest evidence of halogen bond formation was observed in adducts of interhalogen molecules with HMTA. In **7a**, the N...I(Cl) distance is remarkably short (2.267 Å). In **7b**, the N...I(Br) distance is, as expected, a little longer 2.283 Å. Slightly longer N...Br distances of 2.369–2.391 Å were observed in the intriguing compound **7c**, in which two neutral IBr molecules are bonded to one HMTA molecule (Scheme 3). The N···Hal distances in IX (X = Cl, Br) adducts of hexamethylenetetramine show easily understandable trends. The N···I distance in the ICl derivative is slightly shorter than that in the IBr adduct because, probably, of different polarisation of IX bonds. The presence of two IBr molecules attached to the same C_6_H_12_N_4_ unit leads to a strong elongation of the N···Br distances compared to the adduct with one IBr molecule.

The geometry of interhalide-anions appears to be practically linear in all compounds studied. The largest deviation from the linear shape was observed in the case of IBr_2_^−^ anions (Br–I–Br angle decreases up to 173^o^ in **2d**), whereas ICl_2_^−^ and BrCl_2_^−^ moieties show negligible differences from the linear geometry. The interatomic distances and angles for the anionic moieties are listed in Supplementary Table S3.

## Conclusion

In this study, we have prepared several dichloroiodate(I), dibromoiodate(I) and dichlorobromate(I) salts with different mono- and di-basic amine bases. DCI and DBI salts were prepared by an acid-base reaction between preformed HICl_2_ or HIBr_2_ with the respective amine base in acetonitrile. Dihaloiodic acids were synthesized *in situ* by oxidation of elemental iodine (or iodide salt of a quaternary amine) with H_2_O_2_. The single crystals of the compounds were grown and their structures studied with NMR and X-ray diffraction. We observed and characterized strong hydrogen and halogen bonding in non-sterically hindered bases such as HMTA and DABCO. No such phenomena were observed in the case of the strongly sterically hindered base 2,4,6-tri-*tert*-butylpyridine. In addition to the desired products, interesting decomposition products were also observed during the crystallization process forming HMTA salts. Strong hydrogen and halogen bonding together with the accessibility of the non-hindered nitrogen atom promoted the formation of N−X bonds forming compounds **7**.

## Data Availability

The original contributions presented in the study are included in the article/Supplementary Material, further inquiries can be directed to the corresponding authors.
