# Supplementary material for: Oxidation of Iodine to Dihaloiodate(I) Salts of Amines With Hydrogen Peroxides and Their Crystal Structures
Source: Front Chem. 2022 May 5;10:912383. doi: 10.3389/fchem.2022.912383 (PMC9117650; doi:10.3389/fchem.2022.912383)
Supplement: Supplementary file 1 [file DataSheet1.pdf]

Supporting information for

Oxidation of iodine to dihaloiodate(I) salts of amines with hydrogen peroxides and their crystal structures

Griša Grigorij Prinčič[1], Nik Maselj[1], Evgeny Goreshnik[2]*, Jernej Iskra[1]*

[1]Faculty of Chemistry and Chemical Technology, University of Ljubljana, Večna pot 113, 1000, Ljubljana, Slovenia

[2]Department of Inorganic Chemistry and Technology, Jožef Stefan Institute, Jamova 39, 1000, Ljubljana, Slovenia

***Correspondence**
Jernej Iskra:
jernej.iskra@fkkt.uni-lj.si
Evgenij Goreshnik:
Evgeny.Goreshnik@ijs.si

Table of Contents

Experimental 2

Crystal structure data and RAMAN 4

Spectroscopic data for dichloroiodate(I) salts 8

Spectroscopic data for dibromoiodate(I) salts 24

Spectroscopic data for dichlorobromate(I) salts 36

References 38

Experimental

Chemicals and solvents were purchased from commercial sources and used as received. Iodine was powdered in a mortar before use. ^{1}H and ^{13}C NMR spectra were recorded on a Bruker Ascend 600 NMR spectrometer (*Bruker* BioSpin AG, Fällanden, Switzerland). Deuterated chloroform ($CDCl_3$) and deuterated dimethyl sulfoxide (DMSO-d_6) were used as solvents and TMS was used as an internal standard (0 ppm). High resolution mass spectra (HRMS) were obtained on Agilent 6224 Accurate Mass TOF LC/MS system with ESI method (*Agilent*, California, USA). Single-crystal X-ray data for all compounds were collected on a Gemini A diffractometer equipped with an Atlas CCD detector, using graphite monochromated MoKα radiation. The data were treated using the CrysAlisPro software suite program package.[1] Analytical absorption corrections were applied to all data sets. All structures were solved using the dual-space algorithm of the SHELXT[2] program implemented in the Olex crystallographic software.[3] Structure refinement was performed with SHELXL-2014 software.[4]. The figures were prepared using DIAMOND 4.6 software.[5] Crystals of **2d** compound demonstrated repeatable pseudo-merohedral twinning. The twinning law was determined using TwinRotMax procedure implemented in Platon software,[6] reflections from the main domain were separated and final refinement was performed using hklf4 file. CCDC 2163719 (**4d**), 2163720 (**1c**), 2163721 (**1d**), 2163722 (**3b**), 2163723 (**7c**), 2163724 (**5c**), 2163725 (**4c**), 2163726 (**1b**), 2163727 (**7b**) 2167522 (**2c**), 2163724 (**2d**), 2167523 (**7a**), 2167524 (**3c**) contain the supplementary crystallographic data for this paper. This data can be obtained free of charge from The Cambridge Crystallographic Data Centre via www.ccdc.cam.ac.uk/data_request/cif.

Exact concentration of 30% aq. was determined by the iodometric method. MethylDABCO iodide[7], dimethylDABCO[7] iodide and diethylDABCO[8] dibromide were prepared according to literature procedures. Spectroscopic data matched this previously reported. 2,4,6-tri-*tert*-butylpyridine hydrobromide was prepared by dissolving 2,4,6-tri-*tert*-butylpyridine in MeCN and addition of 1 eq. aqueous solution of 48% HBr. Solvent was then removed under reduced pressure to yield a white solid.

Caution. Although we have encountered no difficulties, routine precautions (shields, fume hoods, avoidance of transition metal salts) should be observed whenever possible, as H_2O_2, and hydrogen halides are potentially hazardous compounds.

General procedure for preparation of DCI salts from amines.

Elemental iodine* (127 mg, 0.5 mmol) was dissolved in acetonitrile (5 mL). HCl (37%, 2 eq., 197 mg) was added followed by H_2O_2 (30%, 1 eq., 113 mg). The solution was let stirring at ambient temperature for 3 h after which the color changed from dark brown to yellow. The solution was cooled to 0 °C and to this amine (**1–6a**) was added and the reaction was let to stir for additional 10 min at room temperature. Solvent was distilled off under reduced pressure and the solid was washed with 5 mL of cold ethanol.

*Procedure for preparation of DCI salts form quaternary amine iodides was the same as for amines. Instead of elemental iodine quaternary ammonium salt iodide (1 mmol) was dissolved in 5 mL of acetonitrile. The rest of the procedure was the same.

General procedure for preparation of DBI salts from amines.

Elemental iodine* (127 mg, 0.5 mmol) was dissolved in acetonitrile (5 mL). HBr (48%, 2 eq., 342 mg) was added followed by H_2O_2 (30%, 1 eq., 113 mg). The solution was let to stir at ambient temperature for 10 min after which the color changed from dark brown to orange. The solution was cooled to 0 °C

and to this amine (**1–6a**) was added and the reaction was let to stir for additional 10 min at room temperature. Solvent was distilled off under reduced pressure and the solid was washed with 5 mL of cold ethanol.

*Procedure for preparation of DCI salts form quaternary amine iodides was the same as for amines. Instead of elemental iodine quaternary ammonium salt iodide (1 mmol) was dissolved in 5 mL of acetonitrile. The rest of the procedure was the same.

General procedure for preparation of DCB salts from amine 1a.

1a was prepared according to the method described in this work. 2,4,6-tri-*tert*-butylpyridine hydrobromide (33 mg, 0.1 mmol) was dissolved in DCM (5 mL) and HCl (37%, 1 eq., 10 mg) was added. The solution was cooled to 0 °C and *N*-chlorosuccinimide (13.3 mg, 1 eq.) was added in one portion. The color of the solution changed from transparent to bright yellow. Water (10 mL) was added and the solution extracted to remove the succinimide. Organic phase was separated, dried with anhydrous sodium sulfate and the solvent was evaporated under reduced pressure to yield **1a** (30 mg, 75%).

Crystal structure data and RAMAN

Crystal structures of all intentionally prepared compounds consist of discrete organic cations. ICl_2 Cl $C_6H_{14}N_2$ compound contains two different anions – complex one ICl_2 and chloride-anion. Each of $2ICl_2$, HCl, $C_7H_{15}N_2$ and IBr_2, HBr, $C_7H_{16}N_2$ salts contain also one molecule of corresponding hydrogen halide.

Table 1: Summary of crystal data and refinement results of ICl_2 $C_{17}H_{30}N$, IBr_2 $C_{17}H_{30}N$, $BrCl_2$ $C_{17}H_{30}N$, ICl_2 Cl $C_6H_{14}N_2$, 2(IBr_2) $C_6H_{14}N_2$, ICl_2 $C_6H_{13}N_4$, IBr_2 $C_6H_{13}N_4$, ICl_2, HCl, $C_7H_{15}N_2$ and IBr_2, HBr, $C_7H_{16}N_2$ compounds.

	**1b**	**1c**	**1d**	**2c**	**2d**
Chem. formula	ICl_2 $C_{17}H_{30}N$	IBr_2 $C_{17}H_{30}N$	$BrCl_2$ $C_{17}H_{30}N$	ICl_3 $C_6H_{14}N_2$	2(IBr_2) $C_6H_{14}N_2$
Fw (g/mol)	446.22	535.14	399.23	347.44	687.63
Crystal System	monoclinic	monoclinic	monoclinic	orthorhombic	triclinic
Space group	*P2₁/n*	*P2₁/n*	*P2₁/n*	*P n m a*	$P\ \bar{1}$
a (Å)	10.3401(3)	10.4097(5)	10.1596(8)	14.1145(15)	7.5322(4)
b (Å)	13.8911(4)	14.4148(5)	13.8831(8)	7.5431(6)	14.0963(7)
c (Å)	14.4448(4)	14.4530(6)	14.4795(10)	10.5246(8)	15.4628(7)
α (°)	90	90	90	90	67.605(5)
β (°)	104.229(3)	105.162(4)	104.305(7)	90	77.842(5)
γ (°)	90	90	90	90	88.705(4)
V ($Å^3$)	2011.13(10)	2093.23(16)	1979.0(2)	1120.52(17)	1480.89(14)
Z	4	4	4	4	4
T (K)	150	150	150	150	150
R_1 [a]	0.0247	0.0286	0.0383	0.0380	0.0350
wR_2 [b]	0.0557	0.0624	0.0933	0.0776	0.0919

	**3b**	**3c**	**4c**	**4d**
Chem. formula	ICl_2, $C_6H_{13}N_4$	IBr_2, $C_6H_{13}N_4$	ICl_2, HCl, $C_7H_{15}N_2$	IBr_2, HBr, $C_7H_{16}N_2$
Fw (g/mol)	339.00	427.92	361.47	495.85
Crystal System	monoclinic	triclinic	orthorhombic	monoclinic
Space group	*P2₁/c*	$P\ \bar{1}$	*P n m a*	*P2₁/m*
a (Å)	8.6935(2)	6.4040(4)	14.9012(10)	9.0341(7)
b (Å)	6.3664(2)	10.6322(5)	7.2326(5)	7.7620(6)
c (Å)	19.5868(4)	17.8050(14)	11.6854(10)	9.7196(8)
α (°)	90	99.372(5)	90	90
β (°)	93.698(2)	100.057(6)	90	94.159(7)
γ (°)	90	99.593(5)	90	90
V ($Å^3$)	1081.80(5)	1153.78(13)	1259.39(16)	679.77(9)
Z	4	4	4	2
T (K)	150	150	150	150
R_1 [a]	0.0276	0.0422	0.0236	0.0311
wR_2 [b]	0.0723	0.0935	0.0525	0.0740

[a] $R_1 = \Sigma||F_o| - |F_c||/\Sigma|F_o|$ for $I > 2\sigma(I)$. [b] $wR_2 = [\Sigma[w(F_o^2 - F_c^2)^2]/\Sigma w(F_o^2)^2]^{½}$. [c] Crystal structure determined at 296 K is the same as at 150 K.

Table 2: Summary of crystal data and refinement results of HMTA adducts with ICl and IBr.

	**7a**	**7b**	**7c**
Chem. formula	ICl, $C_6H_{12}N_4$	IBr, $C_6H_{12}N_4$	$(IBr)_2$, $C_6H_{12}N_4$
Fw (g/mol)	302.55	347.01	553.82
Crystal System	monoclinic	monoclinic	monoclinic
Space group	$P2_1/c$	$P2_1/c$	$P2_1/c$
a (Å)	5.9516(4)	5.9830(3)	6.0182(4)
b (Å)	13.6053(8)	13.9188(7)	15.5940(10)
c (Å)	11.9487(7)	12.0407(6)	14.2464(8)
α (°)	90	90	90
β (°)	98.532(6)	98.233(5)	92.276(5)
γ (°)	90	90	90
V (Å^3)	956.82(10)	992.37(9)	1335.94(14)
Z	4	4	4
T (K)	150	150	150
R_1 ^{a}	0.0239	0.0285	0.0392
wR_2 ^{b}	0.0463	0.0554	0.0875

$^aR_1 = \Sigma\|F_o| - |F_c\|/\Sigma|F_o|$ for $I > 2\sigma(I)$. $^bwR_2 = [\Sigma[w(F_o^2 - F_c^2)^2]/\Sigma w(F_o^2)^2]^{½}$. ^{c}Crystal structure determined at 296 K is the same as at 150 K.

The most interesting peculiarities of organic parts geometry is an influense of quaternization. In all HMTA salts C – N distances around protonated nitrogen atoms are markedly elongated: 1.519(4) - 1.528(4) Å against 1.440(4) - 1.477(4) Å for non-protonated nitrogen atoms in **3b**, 1.510(8) - 1.526(8) Å against 1.436(8) - 1.483(8) Å in **3c** respectively. Less pronounced differences were observed in MeDABCO$^+$ moieties: C – N distances are 1.491(5) - 1.506(3) Å for tetra-coordinated nitrogen and 1.491(5) - 1.495(3) Å for three-coordinated in **4c**. Values of 1.476(9) - 1.505(5) Å for tetracoordinated nitrogen atom in **4d** probably suffer from medium orientational disordering of respective cation part, and C – N bonds lengths for three-coordinated N – center are 1.494(8) - 1.496(5) Å. Such a difference between HHMTA$^+$ and MeDABCO$^+$ geometry changes correlate well with well-known instability of HMTA in strongly acidic media.

Table 3: The interatomic distances and angles for anionic parts.

Compound	I – Br	Br – I – Br
**1c**	2.6848(4) - 2.6956(4)	178.82(1)
**2d**	2.6750(8) - 2.7386(8)	173.09(3) - 179.08(3)
**3c**	2.6850(8) - 2.7798(8)	178.20(3) - 178.58(3)
**4d**	2.6771(7) - 2.6909(7)	177.29(3)
	I - Cl	Cl – I - Cl
**1b**	2.5331(6) - 2.5546(6)	178.81(2)
**2c**	2.544(2) - 2.525(2)	178.37(6)
**3b**	2.5445(8) - 2.5563(8)	179.12(2)
**4c**	2.5336(10) - 2.5455(11)	179.19(4)
	Br - Cl	Cl – Br - Cl
**1d**	2.3891(9) - 2.3973(9)	179.07(3)

Table 4: Hydrogen and halogen bonds geometry (Å, °) .

	D—H···*A*	*D*—H	H···*A*	*D*···*A*	*D*—H···*A*
**2c**	N1—H1···Cl3	1.00	2.35	3.097(4)	130.3
**2d**	N1—H1···Br5	1.00	2.61	3.410(6)	136.7
**2d**	N2—H2···Br4	1.00	2.43	3.285(6)	143.3
**2d**	N3—H3···Br8	1.00	2.63	3.369(6)	130.6
**2d**	N4—H4···Br6	1.00	2.42	3.258(6)	140.5
**3b**	N1—H1···Cl2	0.90 (5)	2.46(5)	3.267(3)	150 (3)
**3c**	N1—H1···Br4	0.83(7)	**2.**73(7)	3.385(6)	137(6)
**3c**	N5—H5···Br2	0.76(7)	2.81(7)	3.408(6)	137(7)
**4d**	N8—H8···Br4	1.00	2.16	3.160(5)	177.0
**7a**	N1···I1			2.267(2)	
**7b**	N1···I1			2.288(3)	
**7c**	N1···I1			2.369(6)	
**7c**	N2···I2			2.391(5)	

Geometric parameters of hydrogen bonds (*D*—H, H···*A* and *D*···*A* distances and *D*—H···*A* angles), and halogen bonds (*D*···*A* distances) are listed in Table Sx. In the case of the 2,4,6-tri-*Terc*-butylpyridine derivatives steric hindrances prevent, as it might be expected, formation of hydrogen bonds with the H(N) atom. The N···Cl bond length in the ICl_2^- salt of H_2dabco^{2+} is rather short because it involves isolated Cl^- anion. The N···Br distances in the IBr_2^- analogue vary in rather wide range. In ICl_2^- and IBr_2^- derivatives of mono-protonated hexamethylenetetramine one may note noticeably higher efficiency of N—H···Cl bonding comparitevely to N—H···Br one.

The N···Hal distances in IX (X=Cl, Br) adducts of hexamethylenetetramine show easily understandable trends. The N···I distance in the ICl derivative is slightly shorter than that in the IBr adduct because, probably, of different polarisation of IX bonds. The presence of two IBr molecules attached to the same $C_6H_{12}N_4$ unit leads to a strong elongation of the N···Br distances compared to the adduct with one IBr molecule.

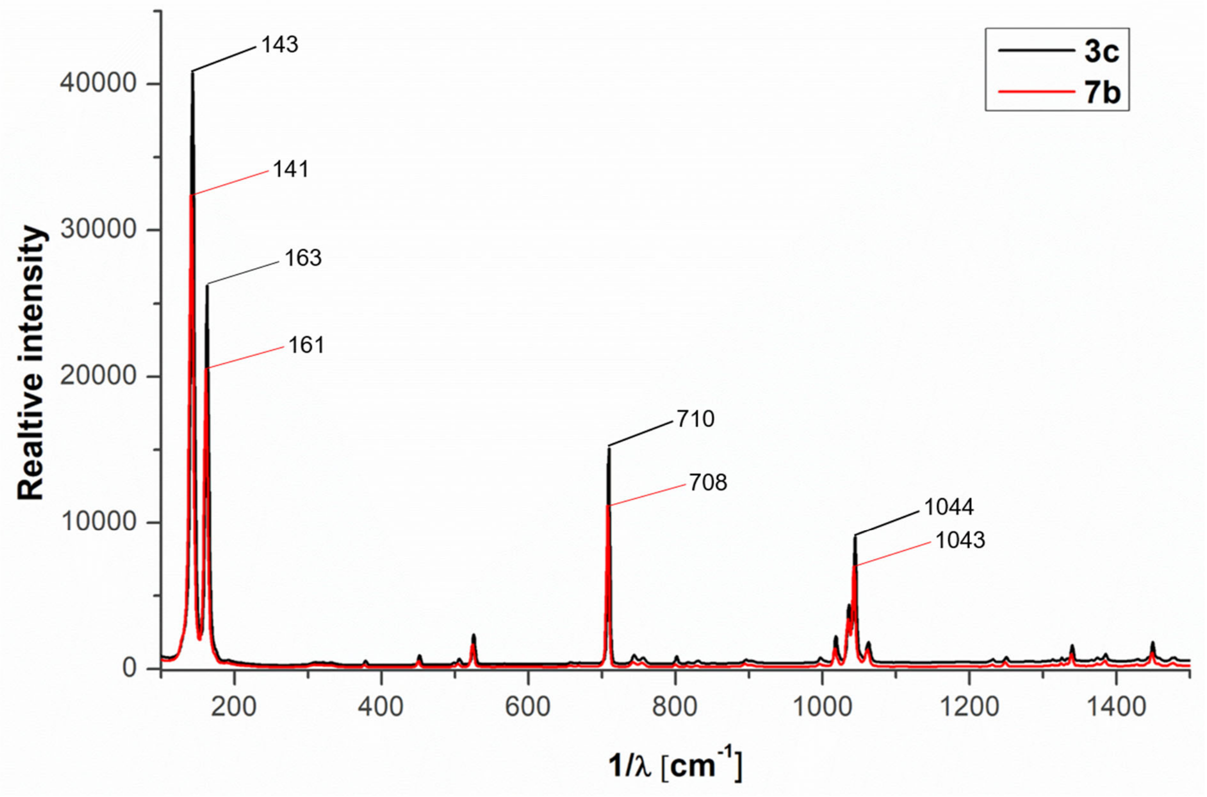

Picture 1: Comparison of RAMAN spectra of **3c** and **7b**.

Spectroscopic data for dichloroiodate(I) salts

2,4,6-Tri-*tert*-butylpyridine dichloroiodate (1b):

Yellow crystalline solid, yield 98 %
^{1}H NMR (600 MHz, DMSO-d_6, 25 °C): δ 1.32 (s, 18H), 1.51 (s, 9H), 7.16 (s, 1.2 H), 7.81 (s, 0.6H), 8.74 (s, 1H).
^{13}C NMR (151 MHz, DMSO-d_6, 25 °C): δ 119.3, 112.7, 37.8, 37.17, 31.0, 30.6, 30.1, 29.1.
HRMS-ESI$^+$: 248.2373 (M+H)$^+$
Raman: 143 cm^{-1}, 265 cm^{-1}.
Crystallization method: **1b** was dissolved in boiling MeOH. The solution was slowly cooled (0.1 K/min) to room temperature. Crystals of **1b** formed in the solution.

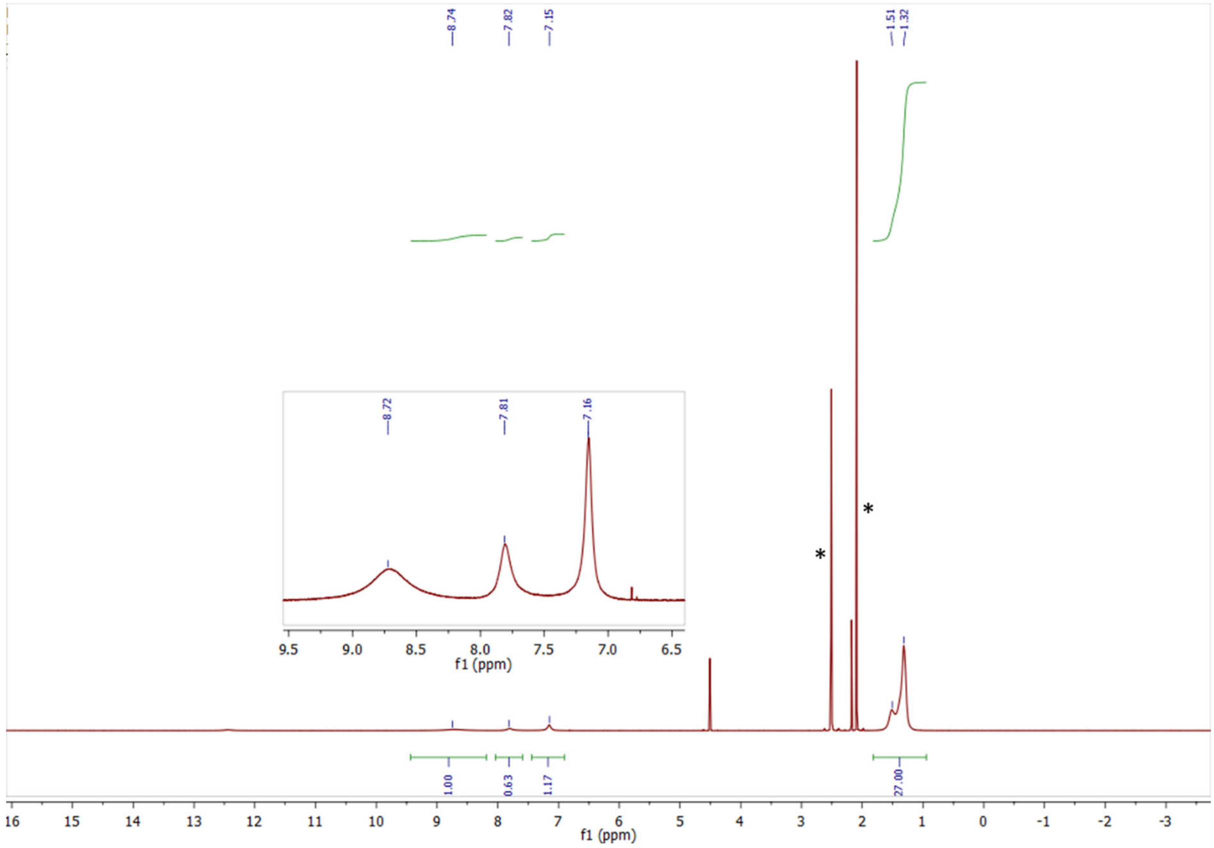

Picture 2: ^{1}H NMR spectrum of **1b** (* denotes residual solvent peaks).

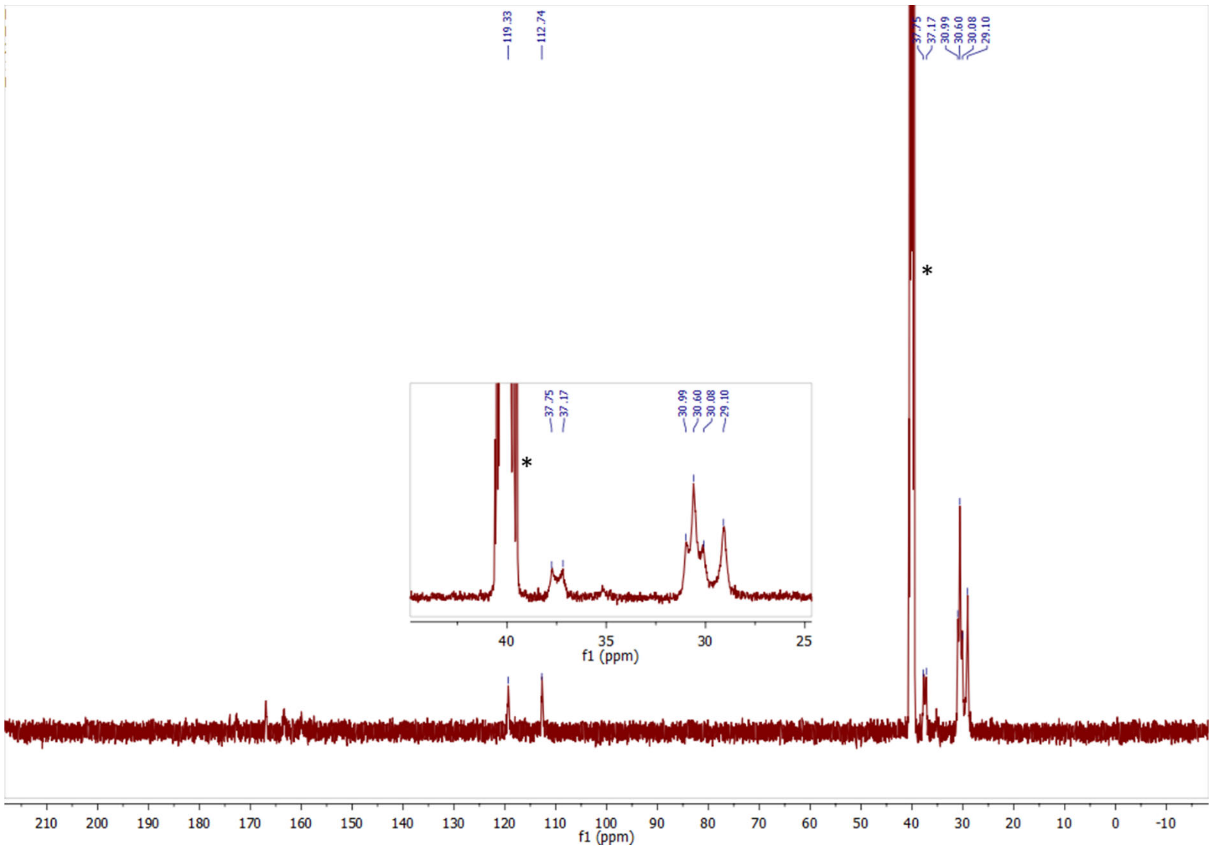

Picture 3: ^{13}C NMR spectrum of 1b (* denotes residual solvent peak).

DABCO bisdichloroiodate(I) (2b):

Yellow crystalline solid, yield 92 %.
^{1}H NMR (600 MHz, DMSO-*d$_6$*, 25 °C): δ 3.47 (s, 12H).
^{13}C NMR (151 MHz, DMSO-*d$_6$*, 25 °C): δ 43.5.
HRMS-ESI^{+}: 113.1074 (M+H)$^{+}$
Crystallization method: **2b** was dissolved in boiling MeCN. The solution was slowly cooled (0.1 K/min) in a dewar flask in the freezer (−20 °C). Crystals of **2b** formed in the solution.

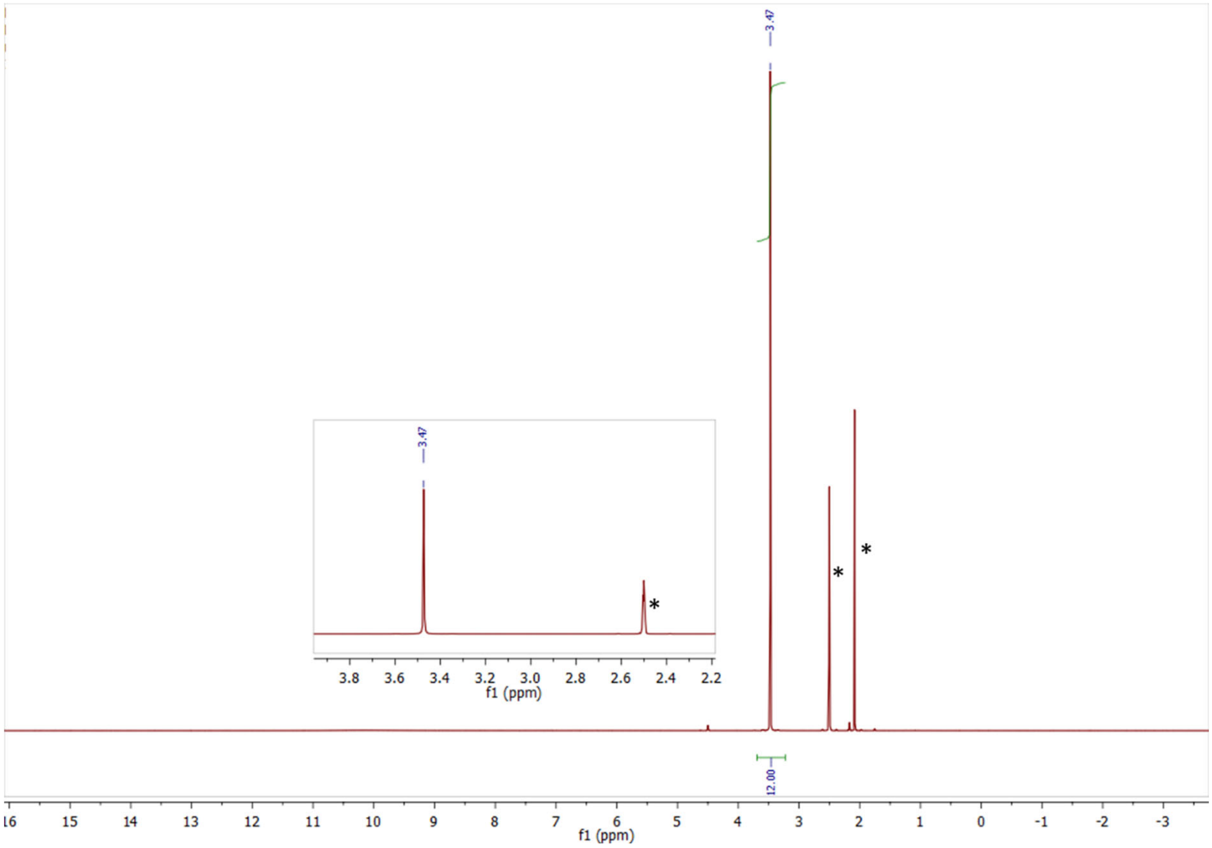

Picture 4: ^{1}H NMR spectrum of **2b** (* denotes residual solvent peaks).

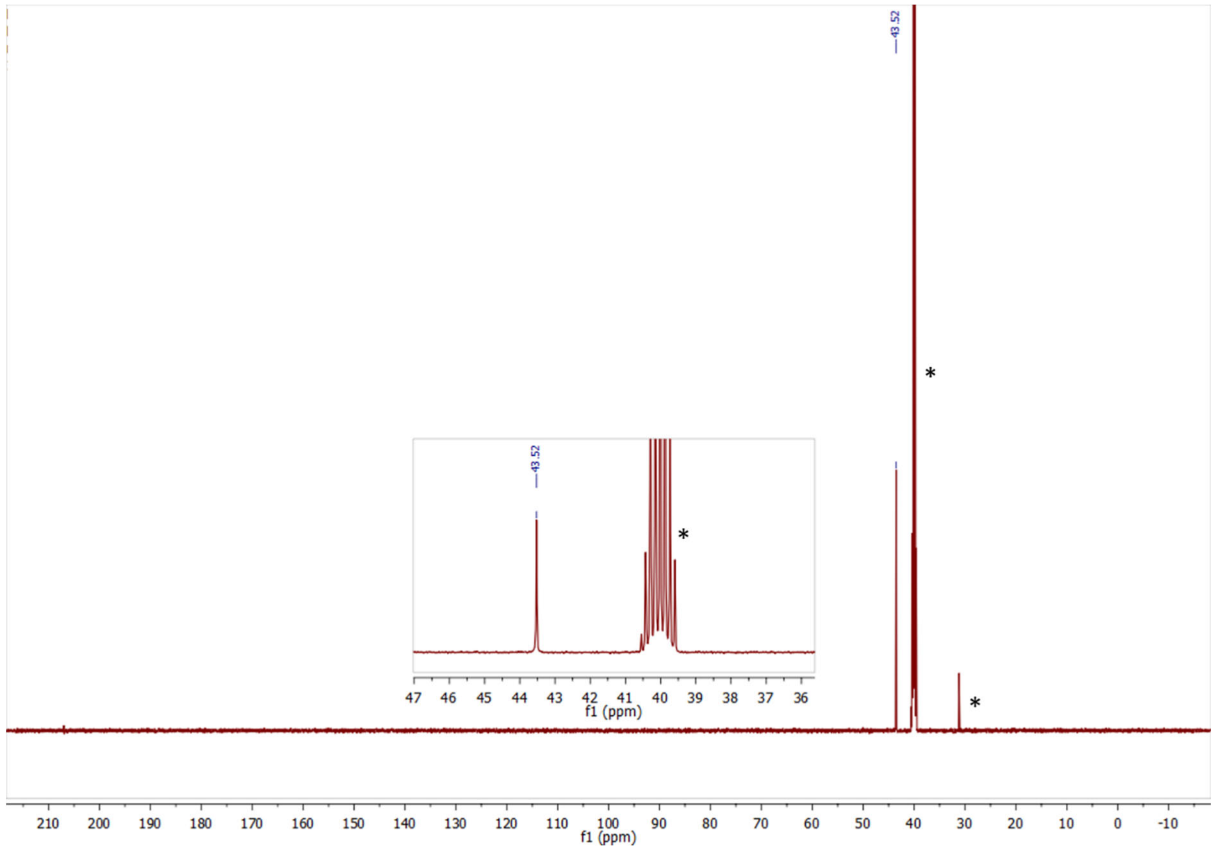

Picture 5: ^{13}C NMR spectrum of **2b** (* denotes residual solvent peaks).

DABCO dichloroiodate(I) chloride (2c):

Yellow crystalline solid, yield 90 %.
^{1}H NMR (600 MHz, DMSO-d_6, 25 °C): δ 3.12 (s, 12H).
^{13}C NMR (151 MHz, DMSO-d_6, 25 °C): δ 44.3.
HRMS-ESI^{+}: 113.1073 (M+H)$^{+}$
Raman: 107 cm^{-1}, 272 cm^{-1}, 800 cm^{-1}, 1036 cm^{-1}.
Crystallization method: **2c** was dissolved in MeCN. The solution was placed in a capped bottle filled with Et_2O. Crystals of **2c** formed in the solution within a week.

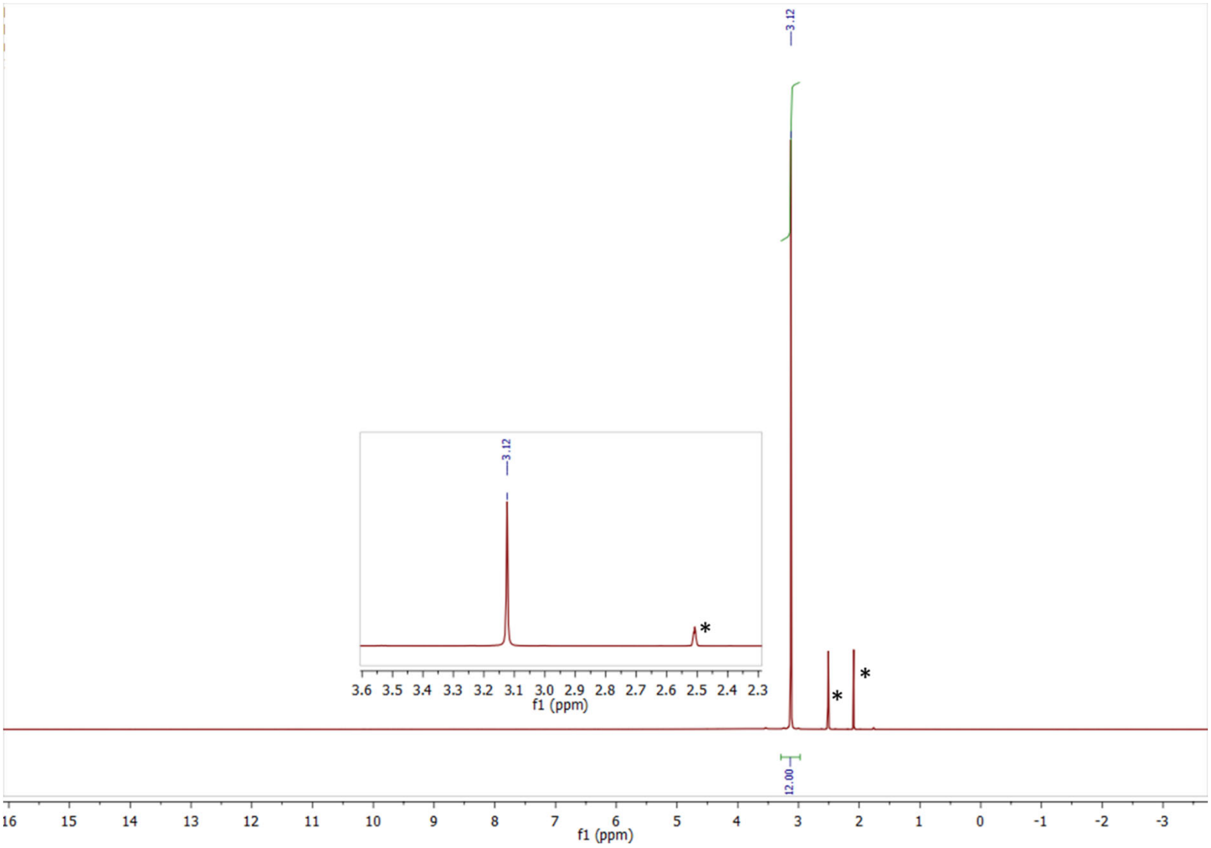

Picture 6: ^{1}H NMR spectrum of **2c** (* denotes residual solvent peaks).

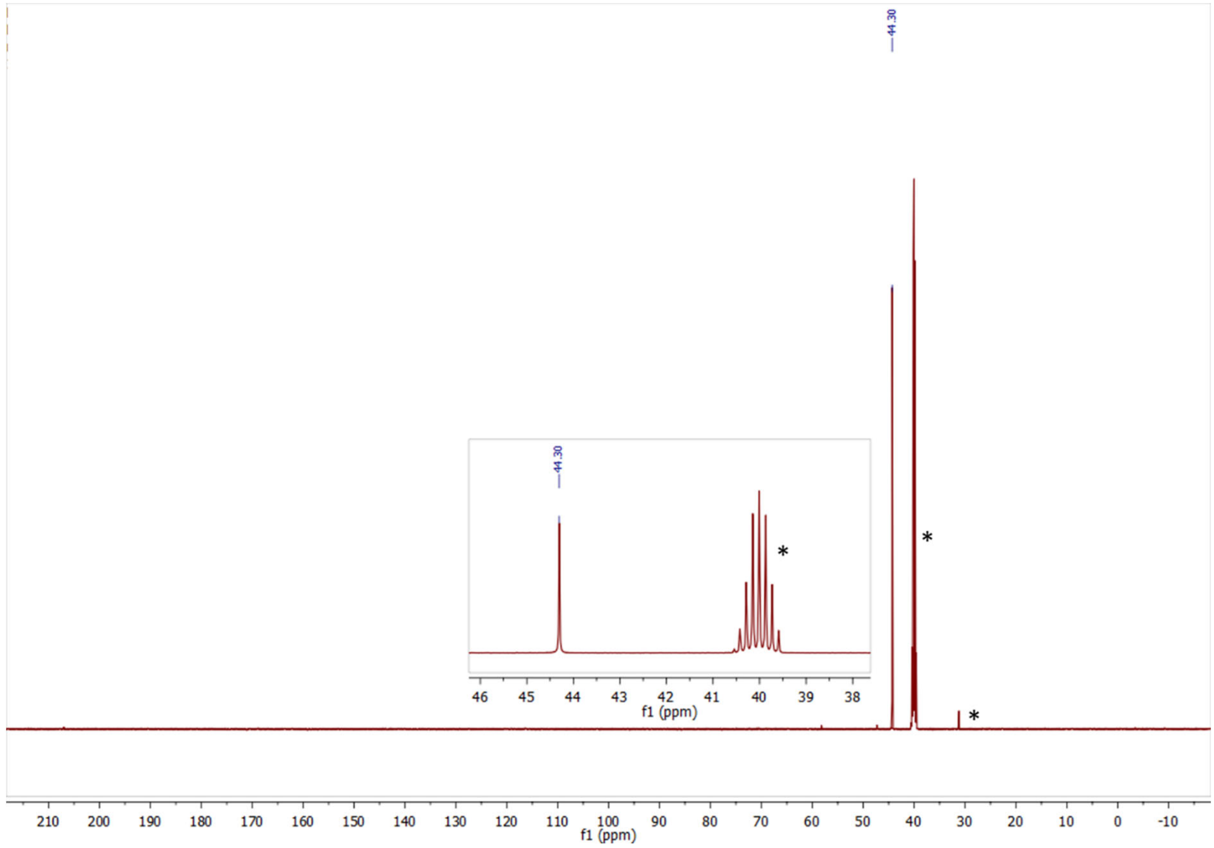

Picture 7: ^{13}C NMR spectrum of **2c** (* denotes residual solvent peaks).

Hexamethylenetetramine dichloroiodate(I) (3b):

Yellow crystalline solid, yield 95 %.
^{1}H NMR (600 MHz, DMSO-d_6, 25 °C): δ 4.81 (s).
^{13}C NMR (151 MHz, DMSO-d_6, 25 °C): δ 72.0.
HRMS-ESI$^+$: 141.1138 $(M+H)^+$
Raman: 152 cm^{-1}, 228 cm^{-1}, 713 cm^{-1}, 1046 cm^{-1}.
Crystallization method: **3b** was dissolved in MeCN/MeOH (v/v 1:1) and the solution was let to evaporate at room temperature. Yellow needle-like crystals of **3b** formed within a week.

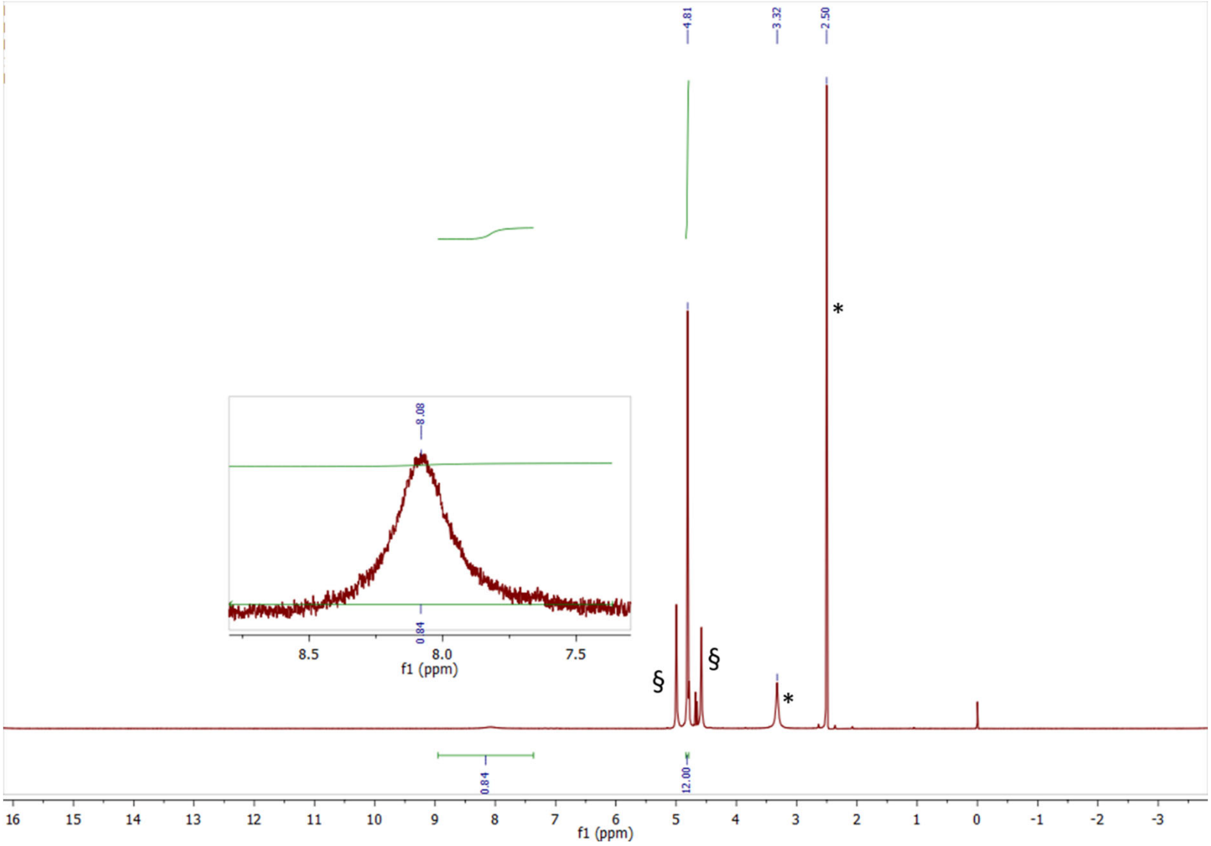

Picture 8: ^{1}H NMR spectrum of **3b** (* denotes water and residual solvent peak, § denotes decomposition products).

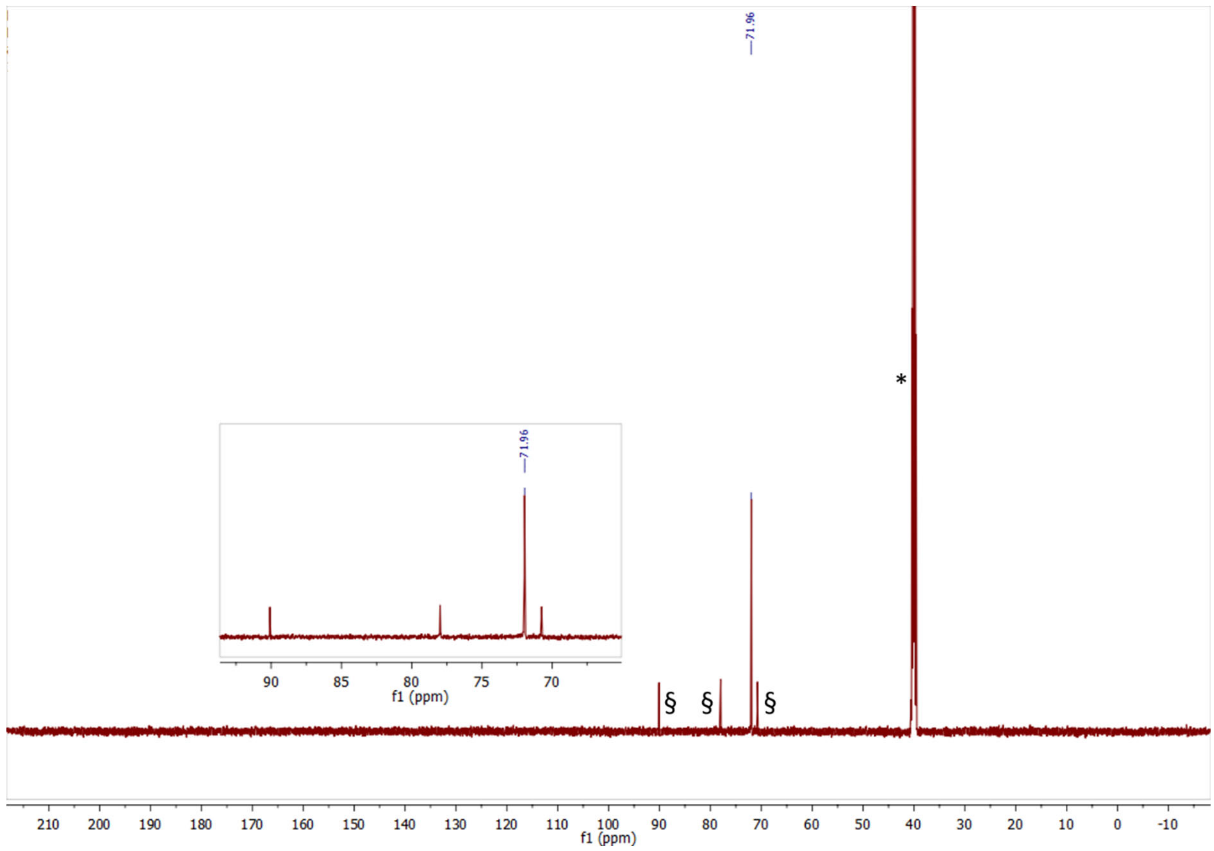

Picture 9: ^{13}C NMR spectrum of **3b** (* denotes residual solvent peak, § denotes decomposition products).

MethylDABCO bisdichloroiodate(I) (4b):

Yellow crystalline solid, yield 86 %
^{1}H NMR (600 MHz, DMSO-d_6, 25 °C): δ 3.11 (s, 3H), 3.39 (t, *J*= 7.6 Hz, 6H), 3.55 (t, J= 7.6 Hz, 6H).
^{13}C NMR (151 MHz, DMSO-d_6, 25 °C): δ 44.2, 51.8, 53.1.
HRMS-ESI^{+}: 127.1229 $(M+H)^{+}$
Crystallization method: **4b** was dissolved in boiling MeOH. The solution was slowly cooled (0.1 K/min) to room temperature. Crystals of **4b** formed in the solution.

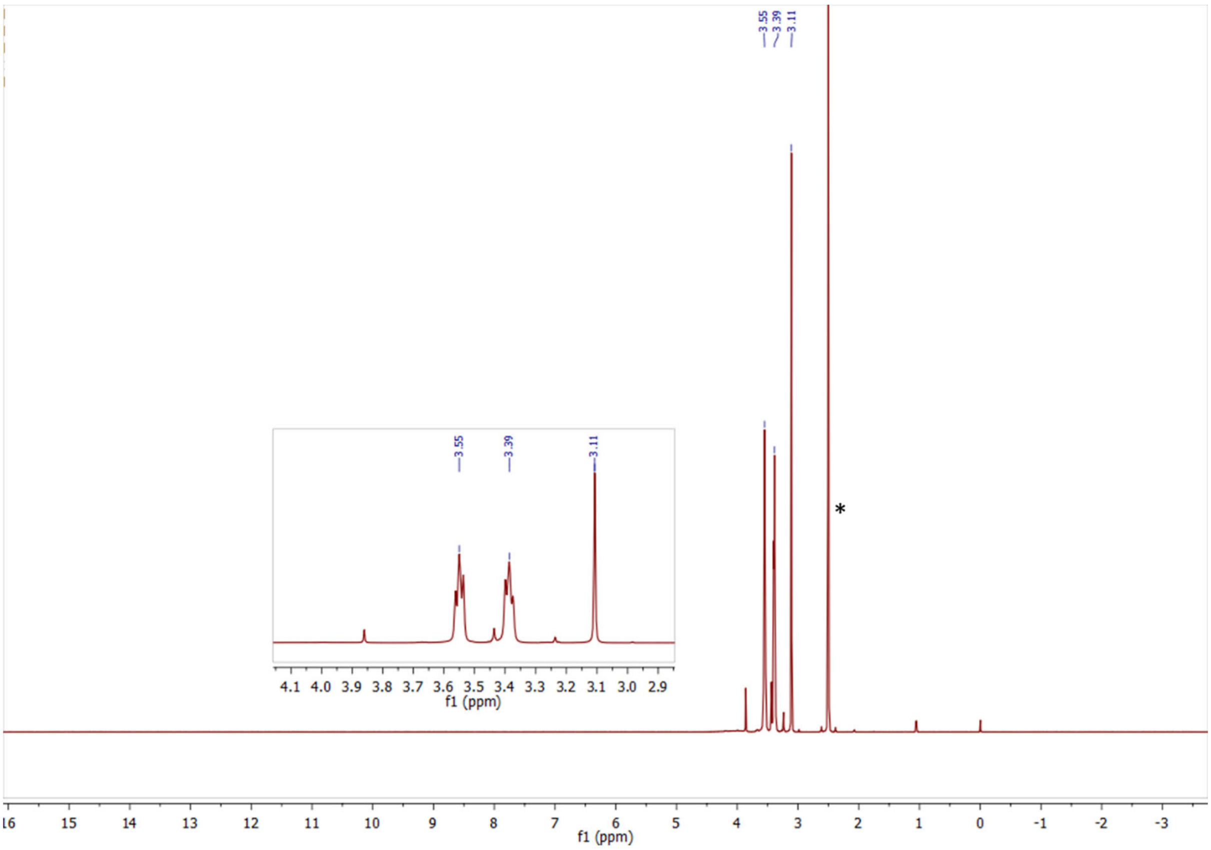

Picture 10: ^{1}H NMR spectrum of **4b** (* denotes residual solvent peak).

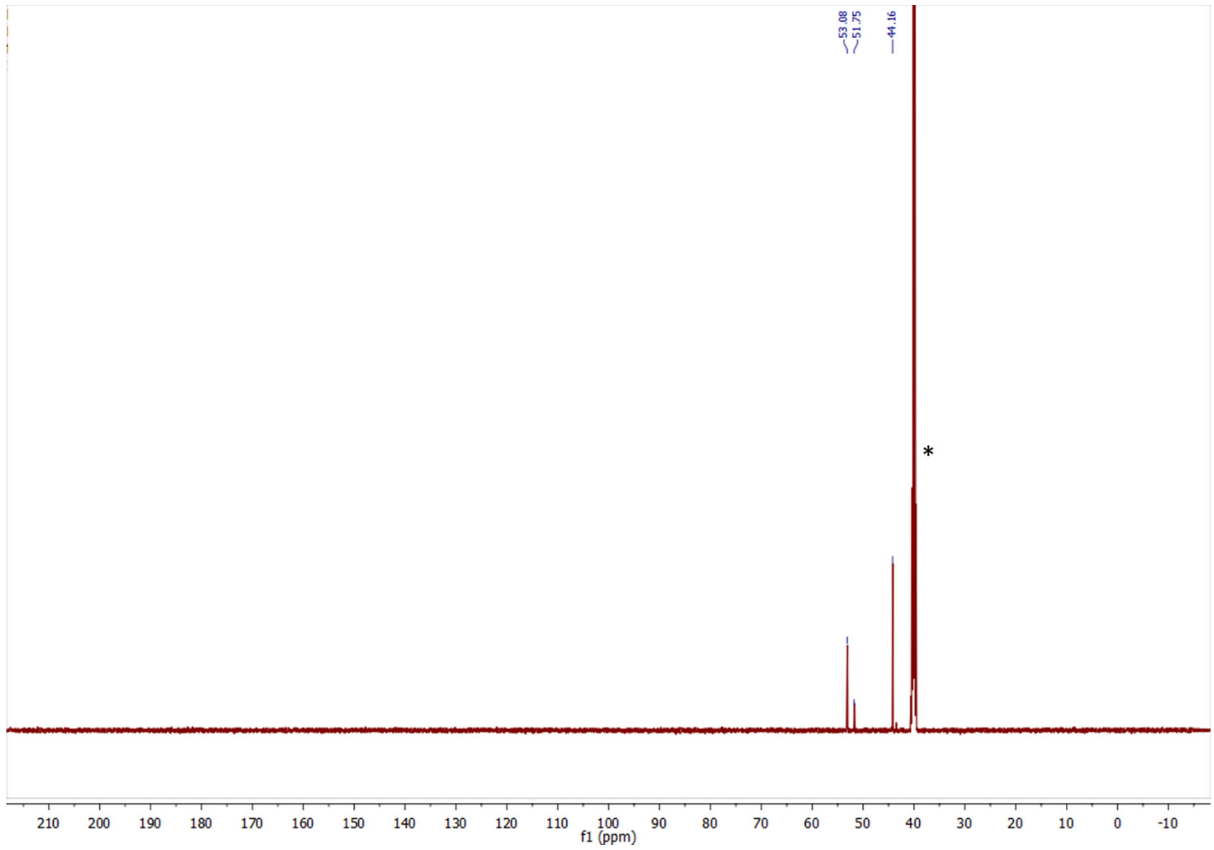

Picture 11: ^{13}C NMR spectrum of **4b** (* denotes residual solvent peak).

MethylDABCO dichloroiodate(I) chloride (4c):

Yellow crystalline solid, yield 87 %.
^{1}H NMR (600 MHz, DMSO-d_6, 25 °C): δ 3.71 – 3.62 (m, 2H), 3.56 – 3.46 (m, 2H), 3.16 (s, 1H)
^{13}C NMR (151 MHz, DMSO-d_6, 25 °C): δ 43.8, 51.8, 52.9.
HRMS-ESI^{+}: 127.1225 $(M+H)^{+}$
Crystallization method: **4c** was dissolved in boiling MeOH. The solution was slowly cooled (0.1 K/min) to room temperature. Crystals of **4c** formed in the solution.

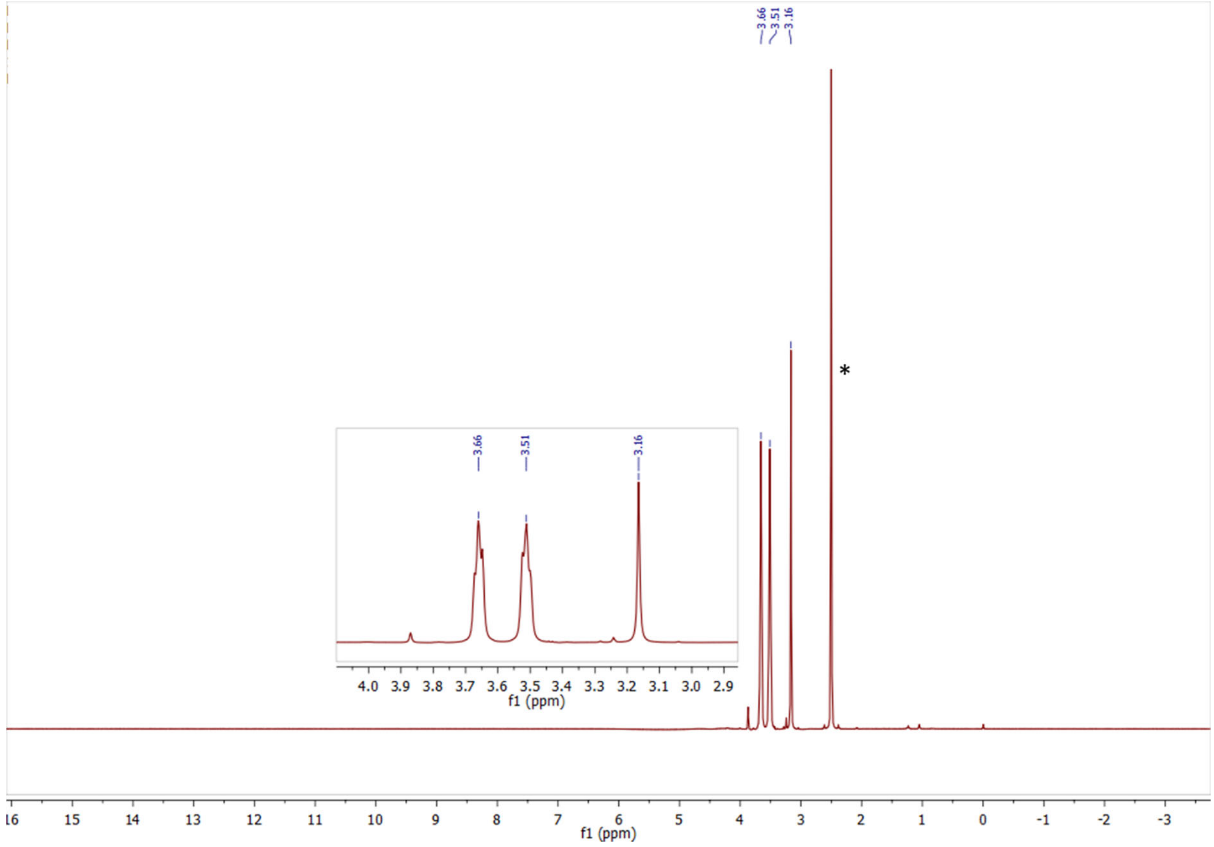

Picture 12: ^{1}H NMR spectrum of **4c** (* denotes residual solvent peak).

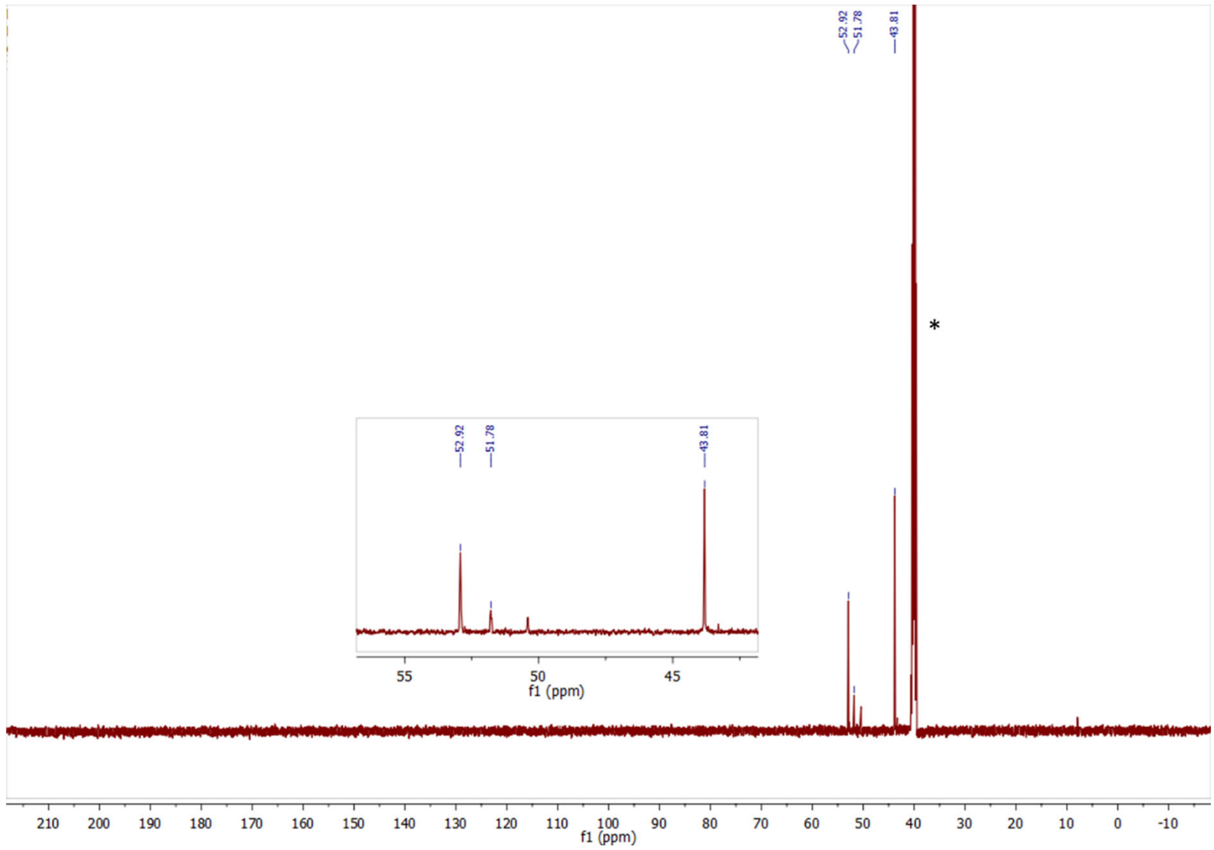

Picture 13: ^{13}C NMR spectrum of **4c** (* denotes residual solvent peak).

DimethylDABCO bisdichloroiodate(I) (5b):

Yellow crystalline solid, yield 96 %
^{1}H NMR (600 MHz, DMSO-d_6, 25 °C): δ 3.23 (s, 6H), 3.85 (s, 12H).
^{13}C NMR (151 MHz, DMSO-d_6, 25 °C): δ 51.9, 52.8.
HRMS-ESI$^+$: 141.1386 (M+H)$^+$
Crystallization method: **5b** was dissolved in MeCN/MeOH (v/v 1:1) and the solution was let to evaporate at room temperature. Yellow needle-like crystals of **5b** formed within a week.

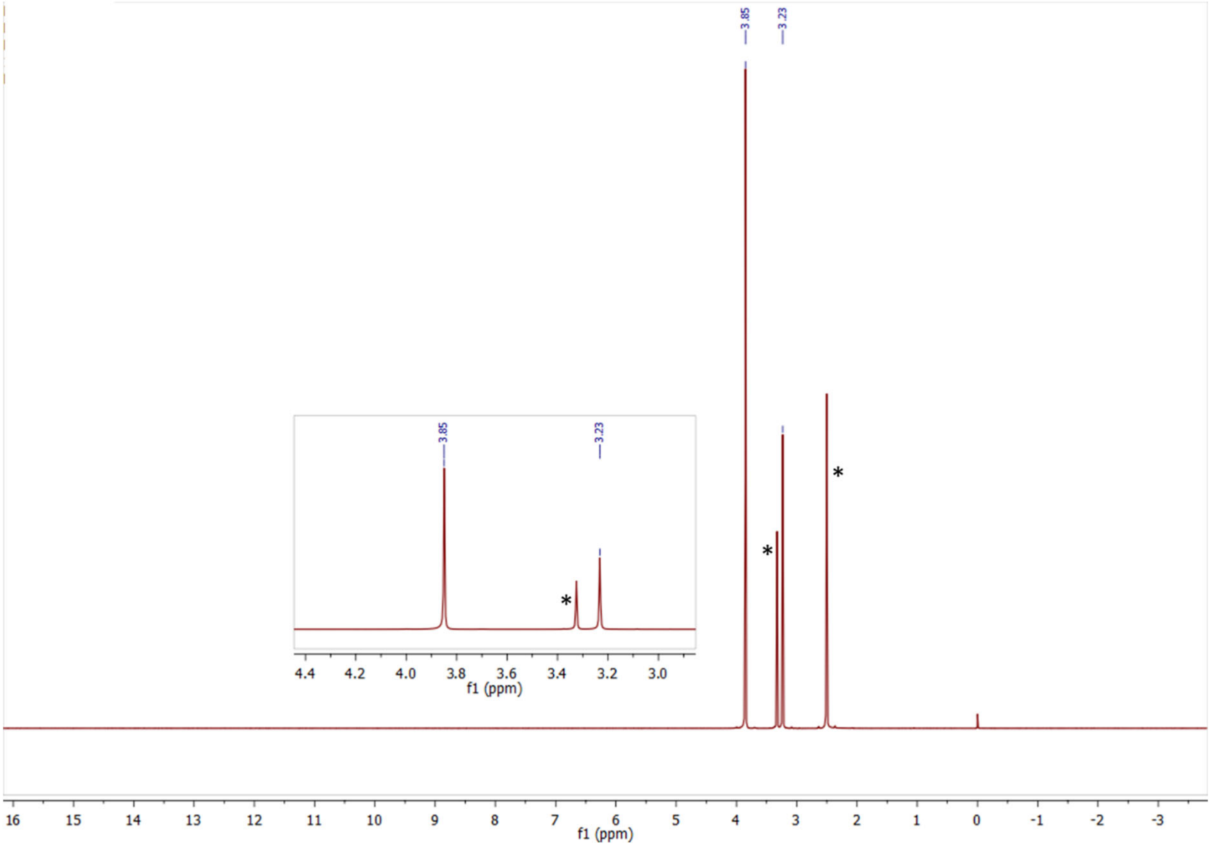

Picture 14: ^{1}H NMR spectrum of **5b** (* denotes water and residual solvent peaks).

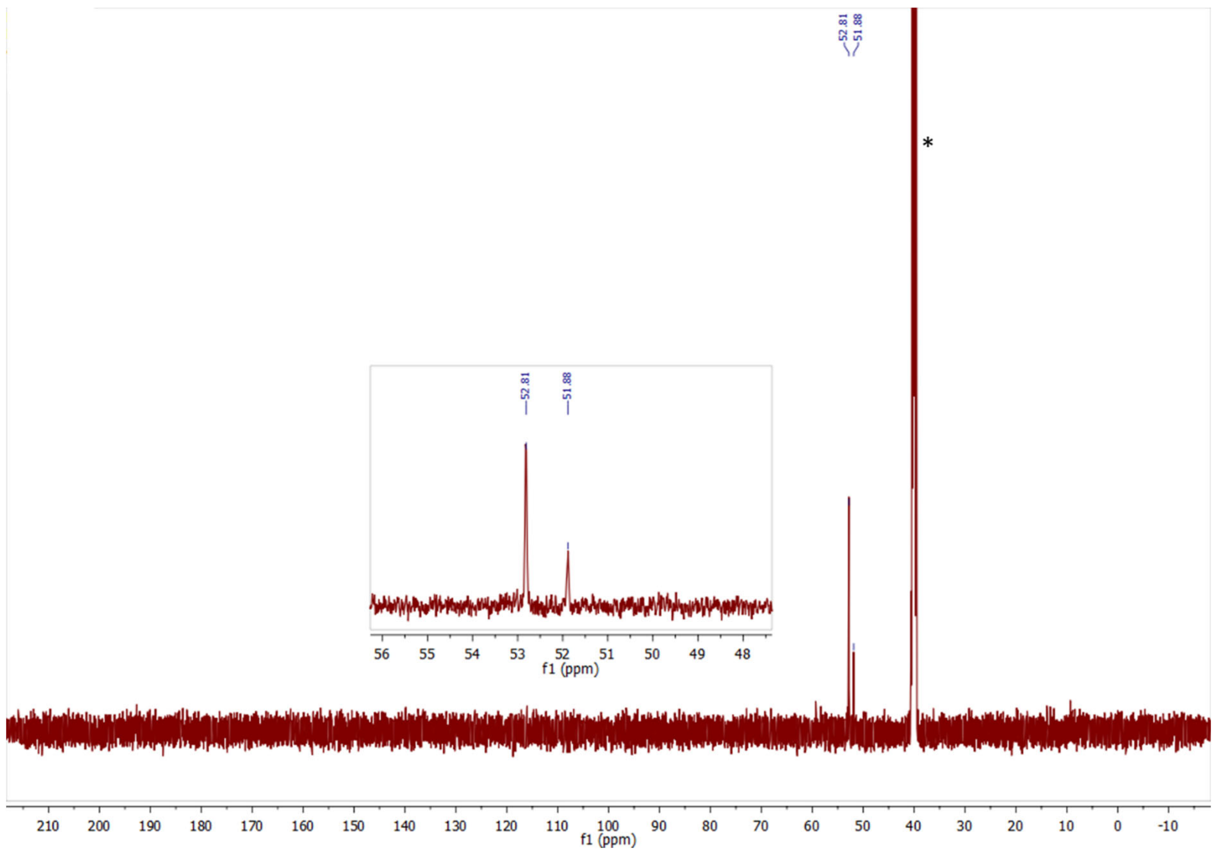

Picture 15: ^{13}C NMR spectrum of **5b** (* denotes residual solvent peak).

DBU dichloroiodate(I) (6b):

Orange sticky crystalline solid, yield 90 %.
^{1}H NMR (600 MHz, DMSO-d_6, 25 °C): δ 9.51 (s, 1H), 3.59 – 3.54 (m, 2H), 3.48 (t, *J* = 5.9 Hz, 2H), 3.26 (td, *J* = 6.1, 3.1 Hz, 2H), 2.69 – 2.60 (m, 2H), 1.93 (p, *J* = 5.9 Hz, 2H), 1.64 (ddt, *J* = 19.9, 9.5, 5.0 Hz, 6H).
^{13}C NMR (151 MHz, DMSO-d_6, 25 °C): δ 165.9, 53.9, 48.4, 38.1, 32.2, 28.7, 26.4, 23.8, 19.3.
HRMS-ESI^{+}: 153.1267 $(M+H)^{+}$
Crystallization method: **6b** was dissolved in MeCN. The solution was placed in a capped bottle filled with Et_2O and into a refrigerator (4 °C). Crystals of **6b** formed in the solution within a week. However, when warmed to room temperature crystals melted/dissolved immediately.

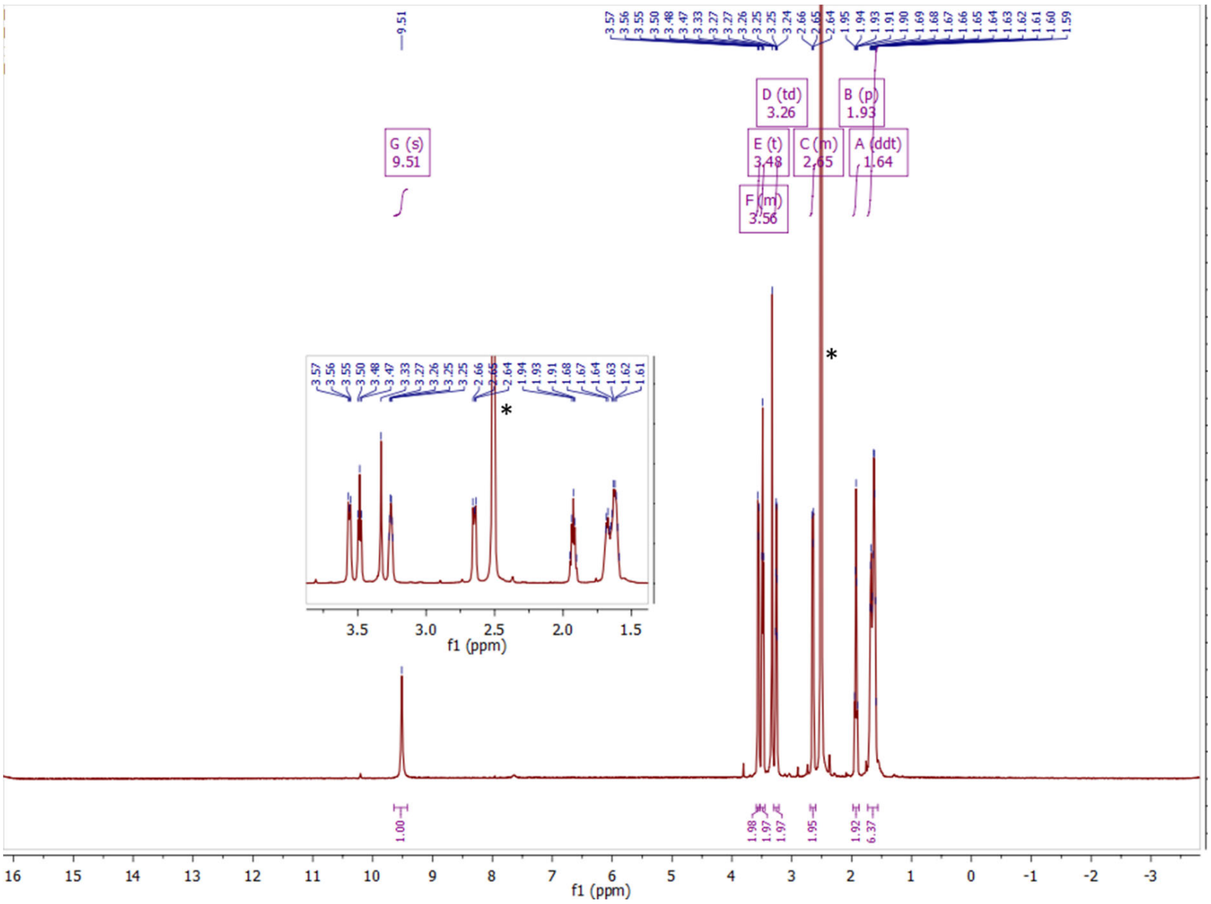

Picture 16: ^{1}H NMR spectrum of **6b** (* denotes residual solvent peaks).

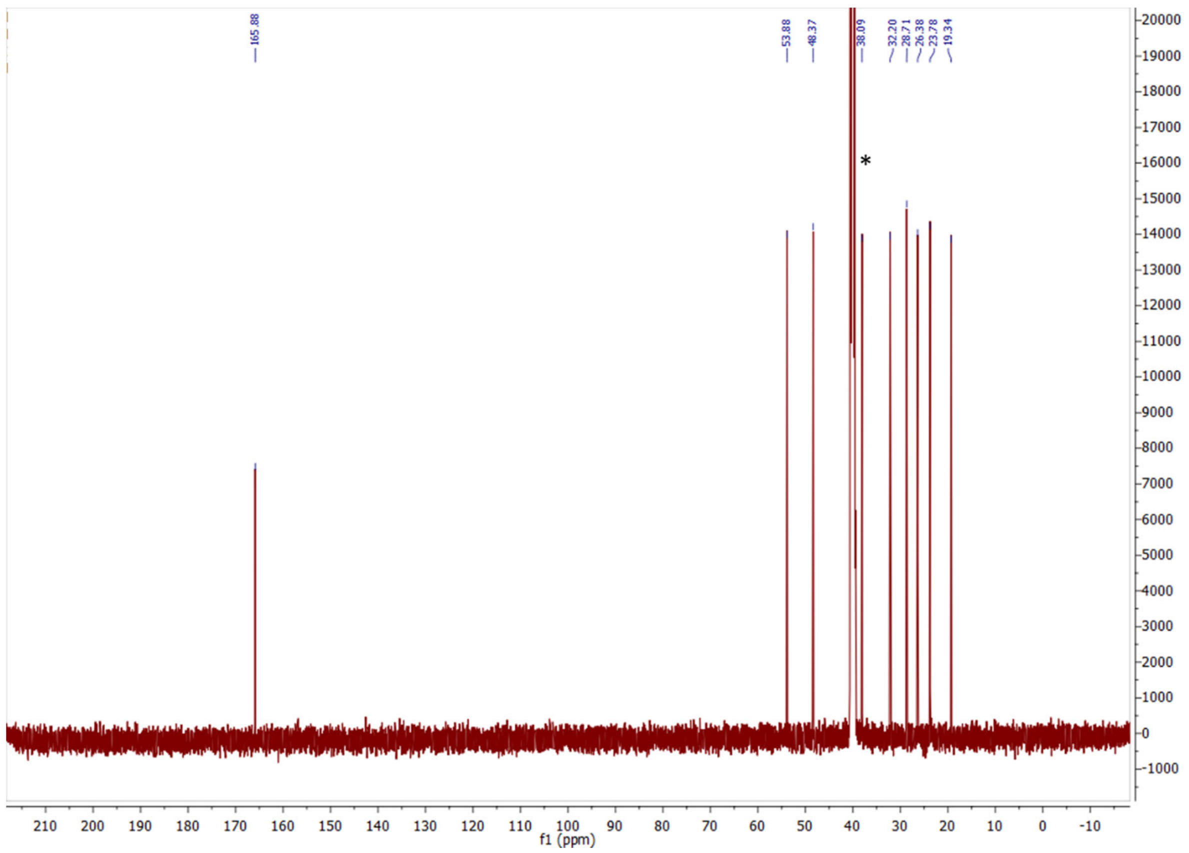

Picture 17: ^{13}C NMR spectrum of **6b** (* denotes residual solvent peaks).

Spectroscopic data for dibromoiodate(I) salts

2,4,6-tri-*tert*-butylpyridine dibromoiodate(I) (1c):

Orange crystalline solid, yield 98 %.
^{1}H NMR (600 MHz, DMSO-d_6, 25 °C): δ 12.39 (s, 0.5H), 11.77 (s, 1H), 7.81 (s, 1H), 7.15 (s, 1H), 1.89 – 1.04 (m, 27H).
^{13}C NMR (151 MHz, DMSO-d_6, 25 °C): δ 119.2, 112.7, 37.7, 37.2, 31.0, 30.6, 30.1, 29.1.
HRMS-ESI$^+$: 248.2373 $(M+H)^+$
Raman: 144 cm^{-1}, 167 cm^{-1}.
Crystallization method: **1c** was dissolved in MeCN/MeOH (v/v 1:1). The solution was placed in a capped bottle filled with Et_2O and into a refrigerator (4 °C). Crystals of **1c** formed in the solution within a week.

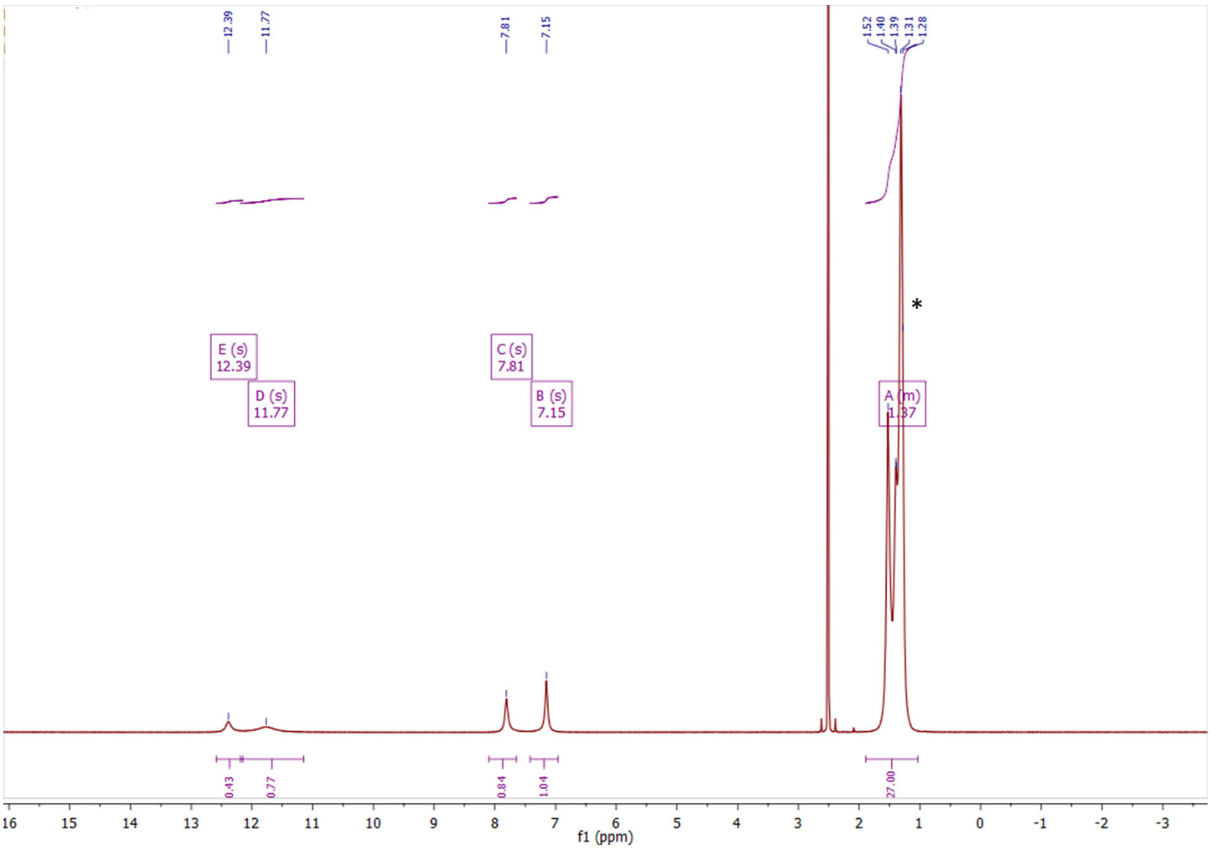

Picture 18: ^{1}H NMR spectrum of **1c** (* denotes residual solvent peak).

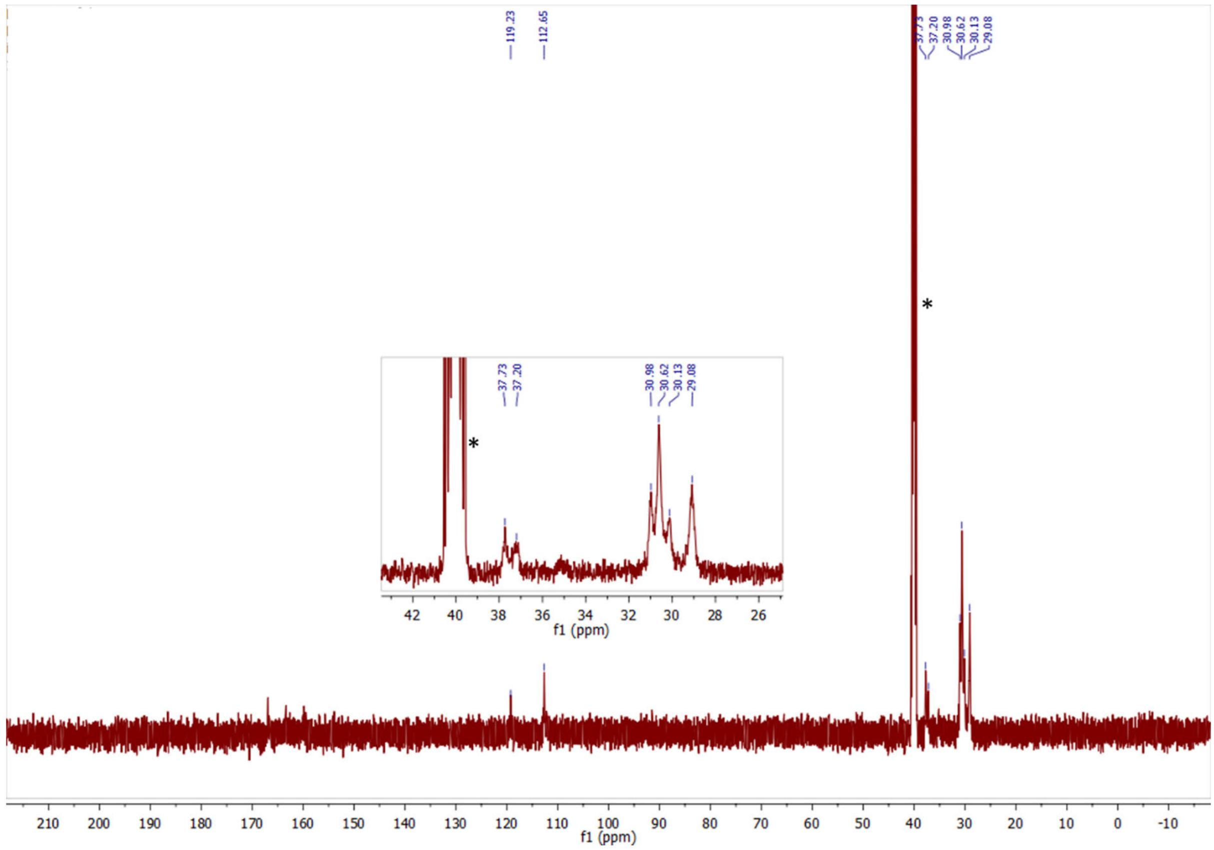

Picture 19: ^{13}C NMR spectrum of **1c** (* denotes residual solvent peak).

DABCO bisdibromoiodate(I) (2d):

Red crystalline solid, yield 88 %.
^{1}H NMR (600 MHz, DMSO-*d*$_6$, 25 °C): δ 3.5 (s, 12H).
^{13}C NMR (151 MHz, DMSO-*d*$_6$, 25 °C): δ 43.5.
HRMS-ESI^{+}: 113.1073 $(M+H)^{+}$
Raman: 162 cm^{-1}, 169 cm^{-1}, 178 cm^{-1}.
Crystallization method: **2d** was dissolved in MeCN/MeOH (v/v 1:1). The solution was placed in a capped bottle filled with Et_2O and into a refrigerator (4 °C). Crystals of **2d** formed in the solution within a week.

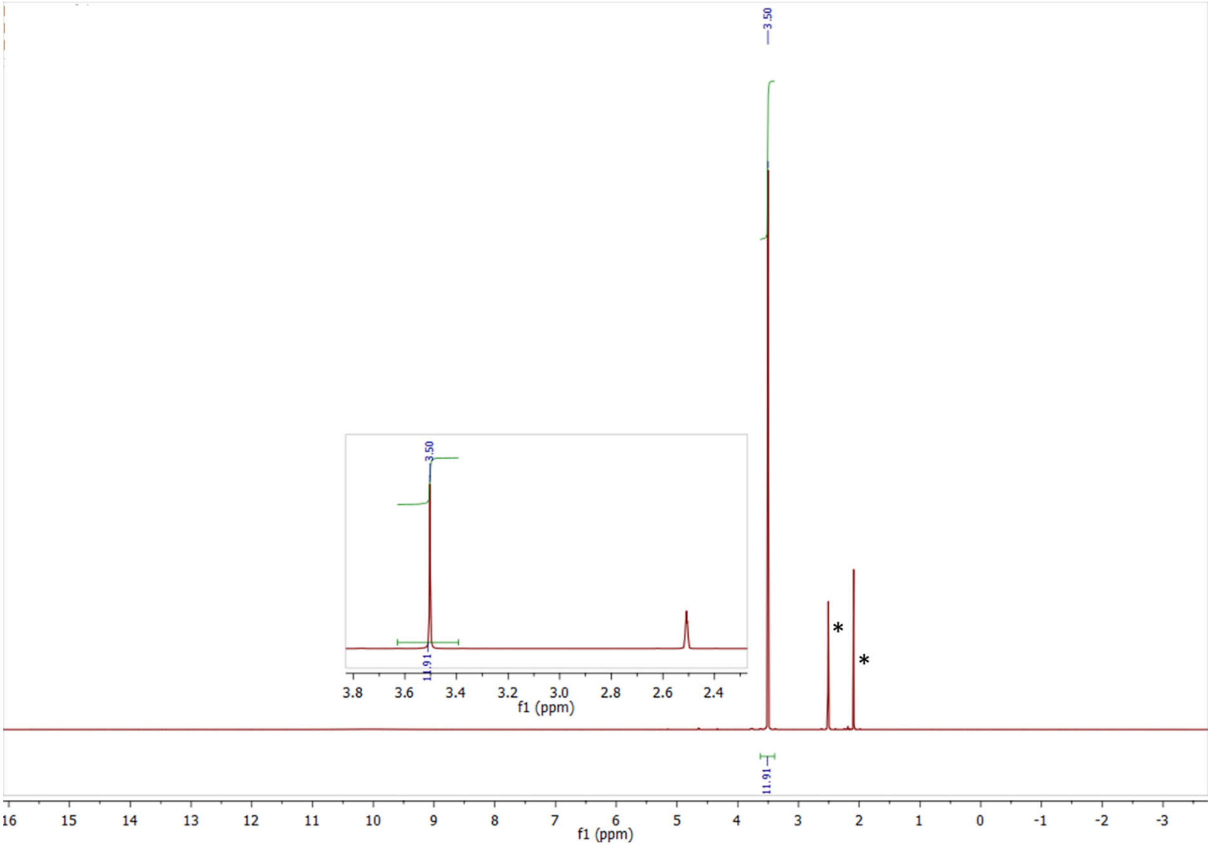

Picture 20: ^{1}H NMR spectrum of **2d** (* denotes residual solvent peak).

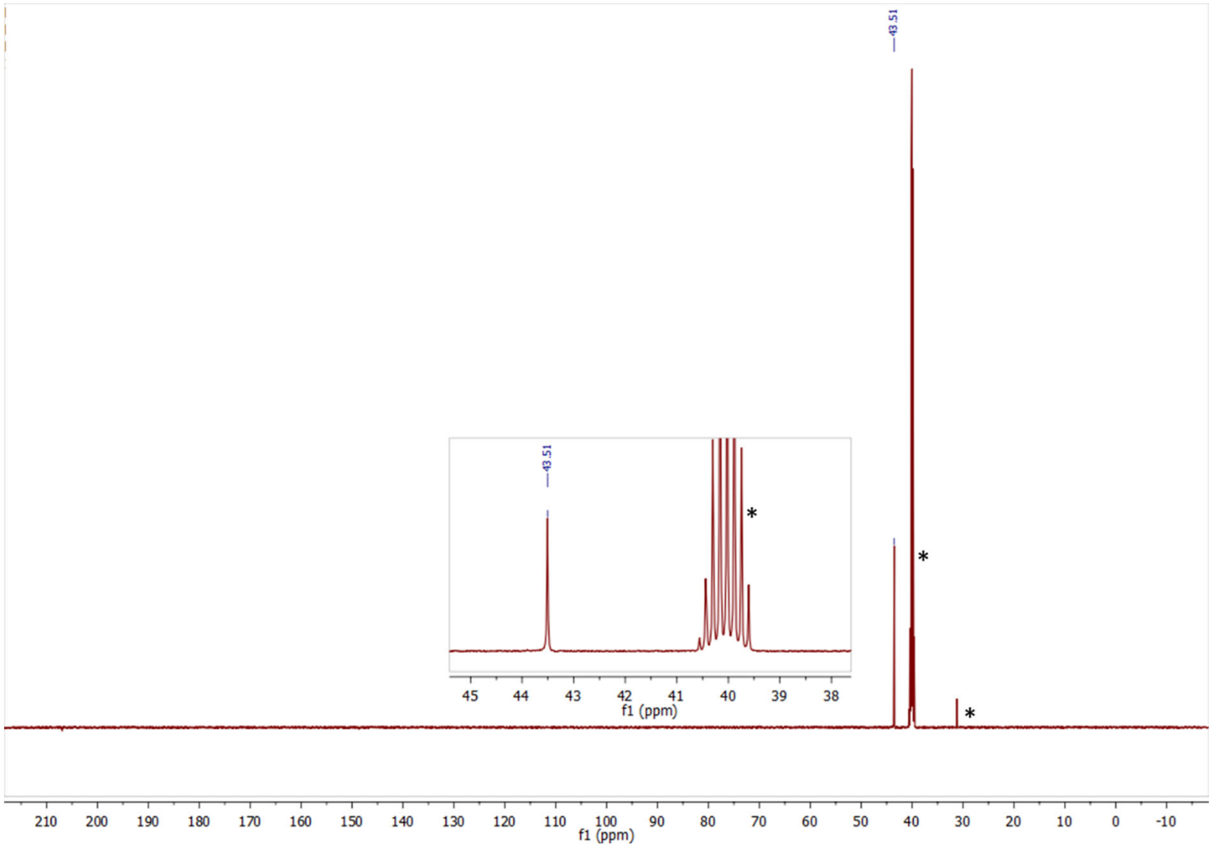

Picture 21: ^{13}C NMR spectrum of **2d** (* denotes residual solvent peak).

Hexamethylenetetramine dibromoiodate(I) (3c):

Orange crystalline solid, yield 93 %.
^1H NMR (600 MHz, DMSO-d_6, 25 °C): δ 4.82 (s, 12H).
^{13}C NMR (151 MHz, DMSO-d_6, 25 °C): δ 72.8.
HRMS-ESI$^+$: 141.1135 $(M+H)^+$
Raman: 143 cm^{-1}, 163 cm^{-1}, 710 cm^{-1}, 1044 cm^{-1}.
Crystallization method: **3c** was dissolved in MeCN/DMSO (v/v 1:1). The solution was placed in a capped bottle filled with Et_2O and into a refrigerator (4 °C). Crystals of **3c** formed in the solution within a week.

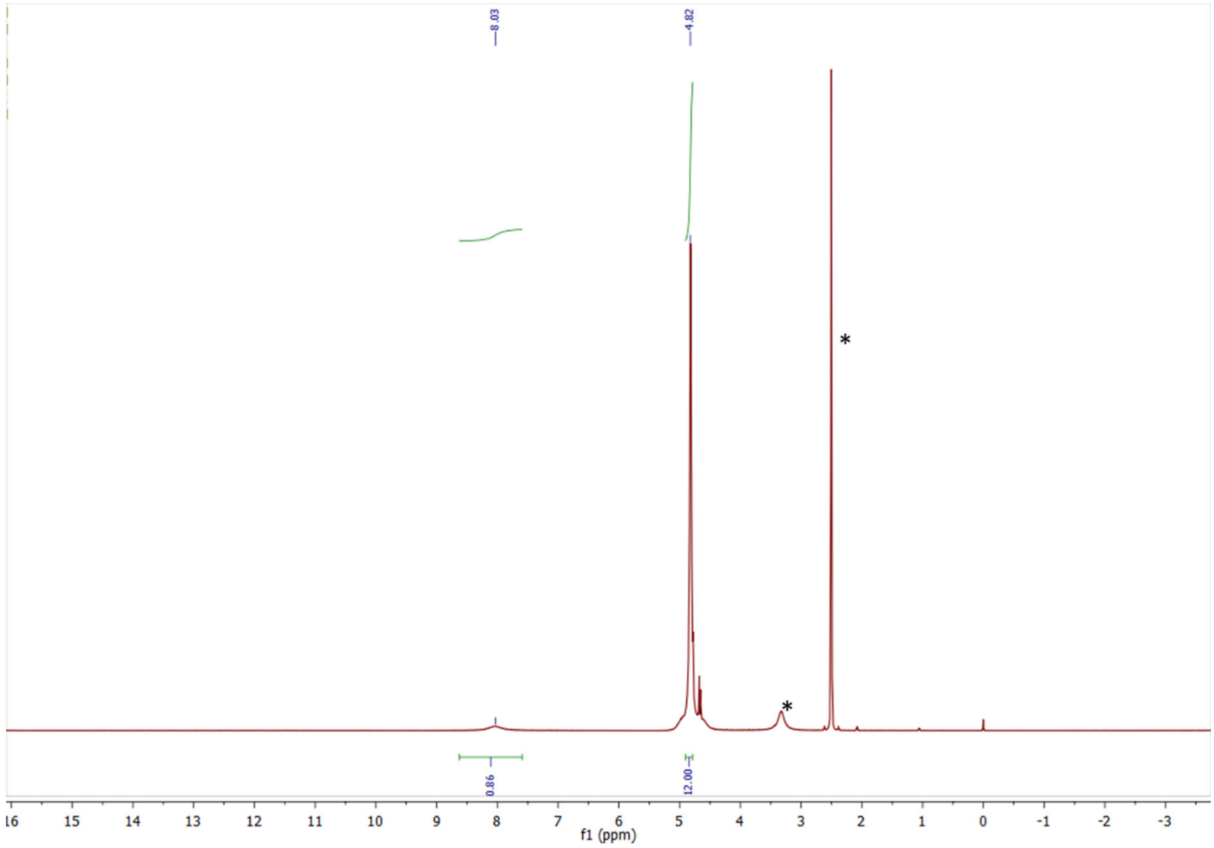

Picture 22: ^1H NMR spectrum of **3c** (* denotes residual solvent peak and H_2O).

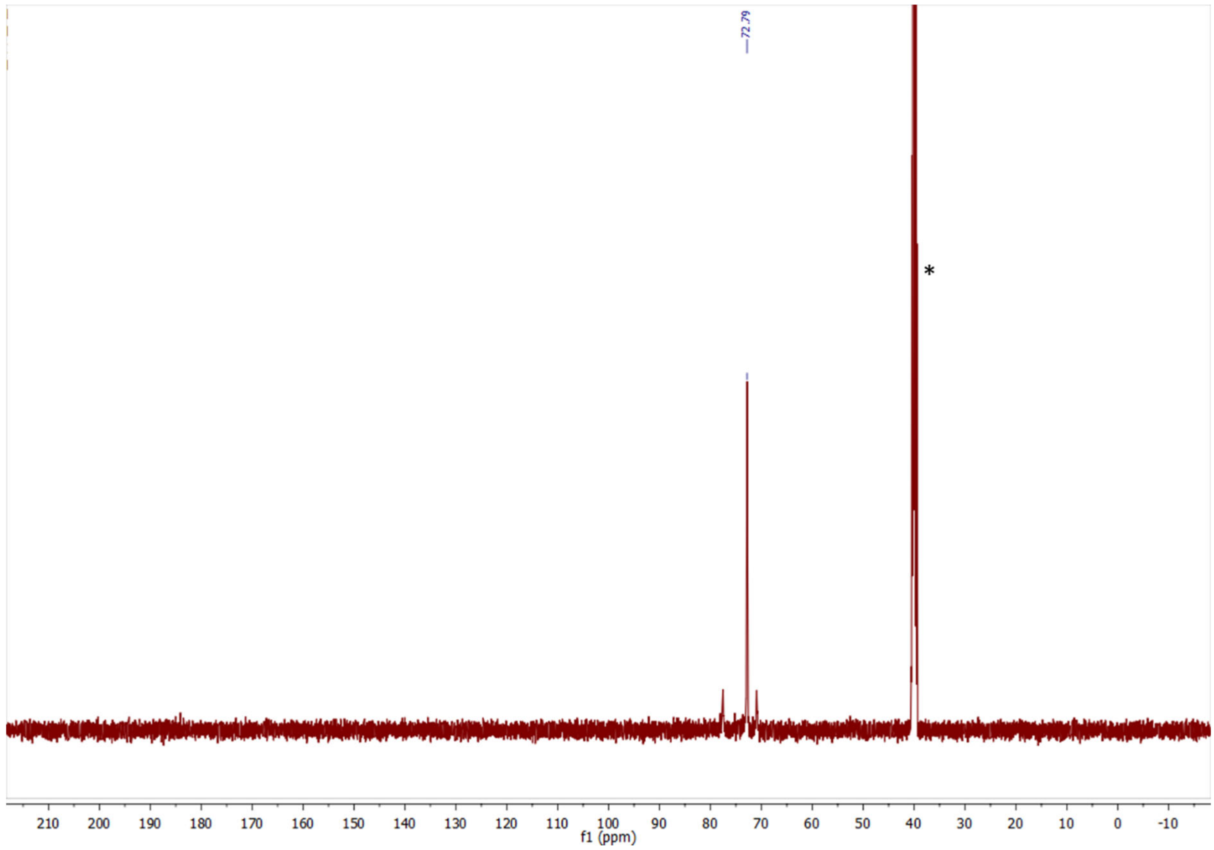

Picture 23: ^{13}C NMR spectrum of **3c** (* denotes residual solvent peak).

MethylDABCO dibromoiodate(I) bromide (4d):

Orange crystalline solid, yield 87 %.

^{1}H NMR (600 MHz, DMSO-d_6, 25 °C): δ 3.67 – 3.59 (m, 6H), 3.53 – 3.45 (m, 6H), 3.15 (s, 3H).

^{13}C NMR (151 MHz, DMSO-d_6, 25 °C): δ 52.9, 51.8, 44.1.

HRMS-ESI$^+$: 127.1231 $(M+H)^+$

Crystallization method: **4d** was dissolved in MeCN/MeOH (v/v 1:1). The solution was placed in a capped bottle filled with Et_2O and into a refrigerator (4 °C). Crystals of **4d** formed in the solution within a week.

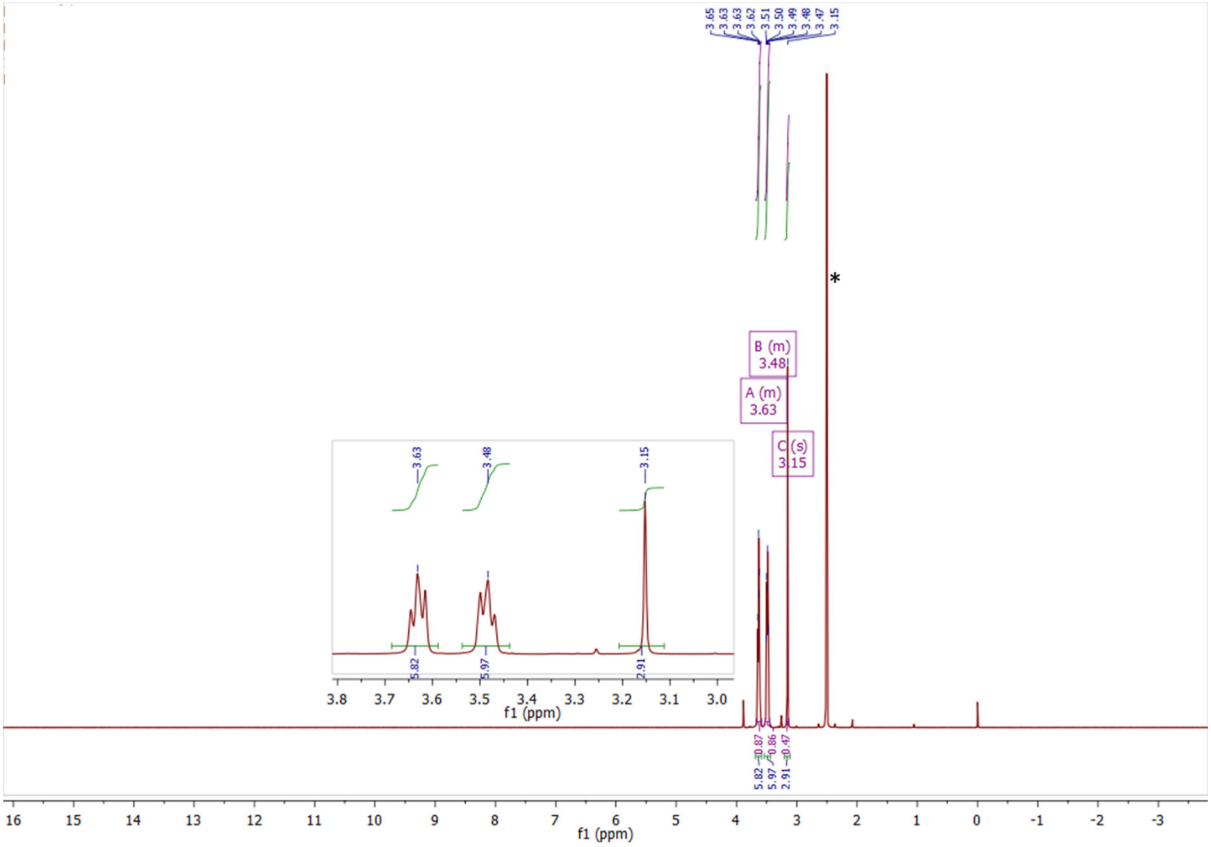

Picture 24: ^{1}H NMR spectrum of **4d** (* denotes residual solvent peak).

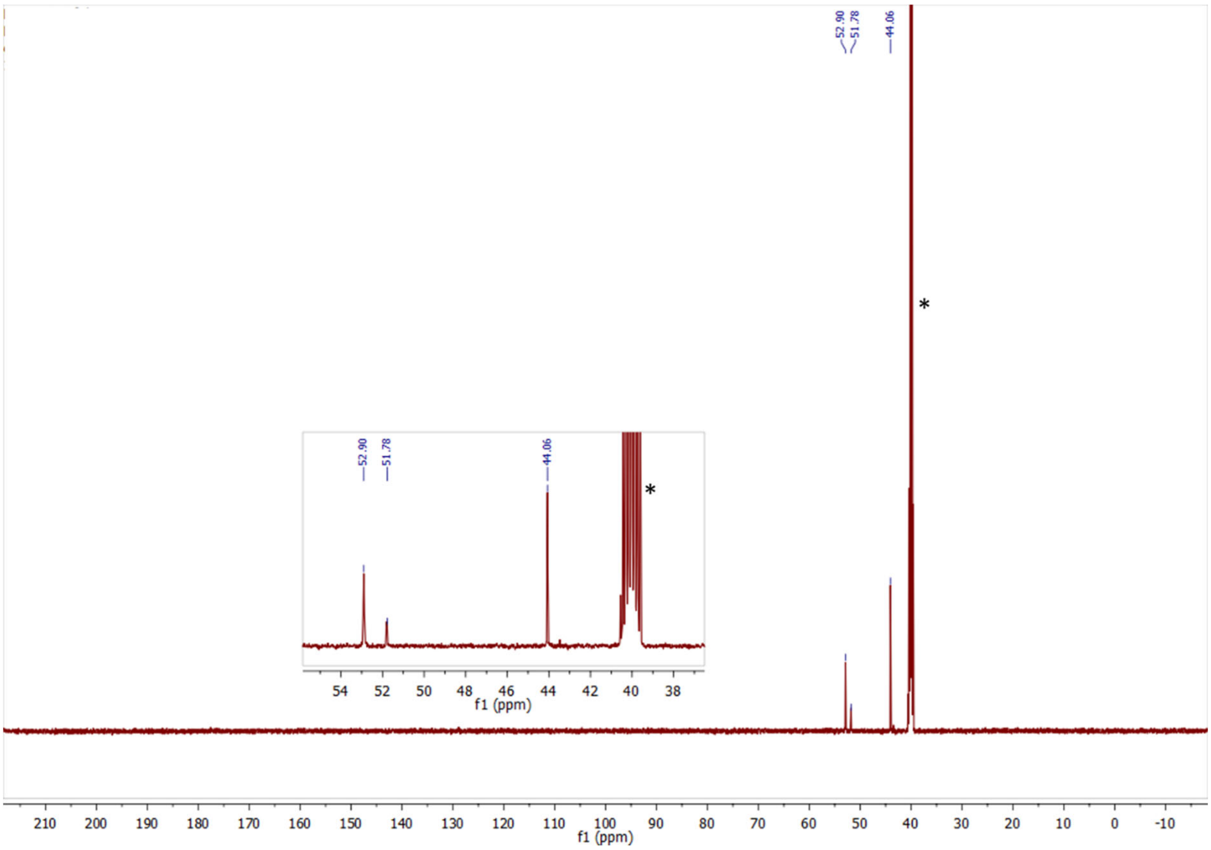

Picture 25: ^{13}C NMR spectrum of **4d** (* denotes residual solvent peak).

DimethylDABCO bisbromoiodate(I) (5c):

Orange crystalline solid, yield 92 %.
^{1}H NMR (600 MHz, DMSO-d_6, 25 °C): δ 3.24 (s, 6H), 3.86 (s, 12H).
^{13}C NMR (151 MHz, DMSO-d_6, 25 °C): δ 51.9, 52.8.
HRMS-ESI$^+$: 141.1390 $(M+H)^+$
Raman: 164 cm^{-1}.
Crystallization method: **5c** was dissolved in MeCN/MeOH (v/v 1:1). The solution was placed in a capped bottle filled with Et_2O and into a refrigerator (4 °C). Crystals of **5c** formed in the solution within a week.

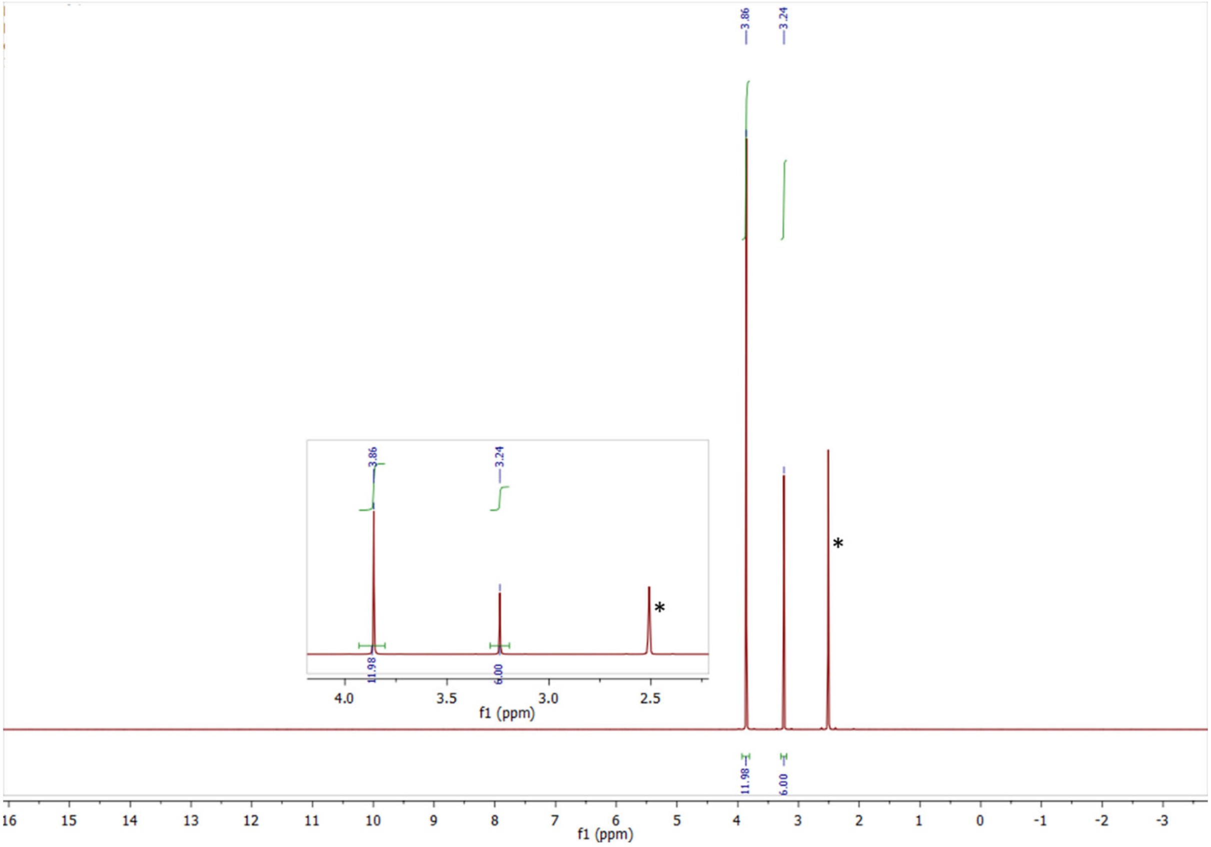

Picture 26: ^{1}H NMR spectrum of **5c** (* denotes residual solvent peak).

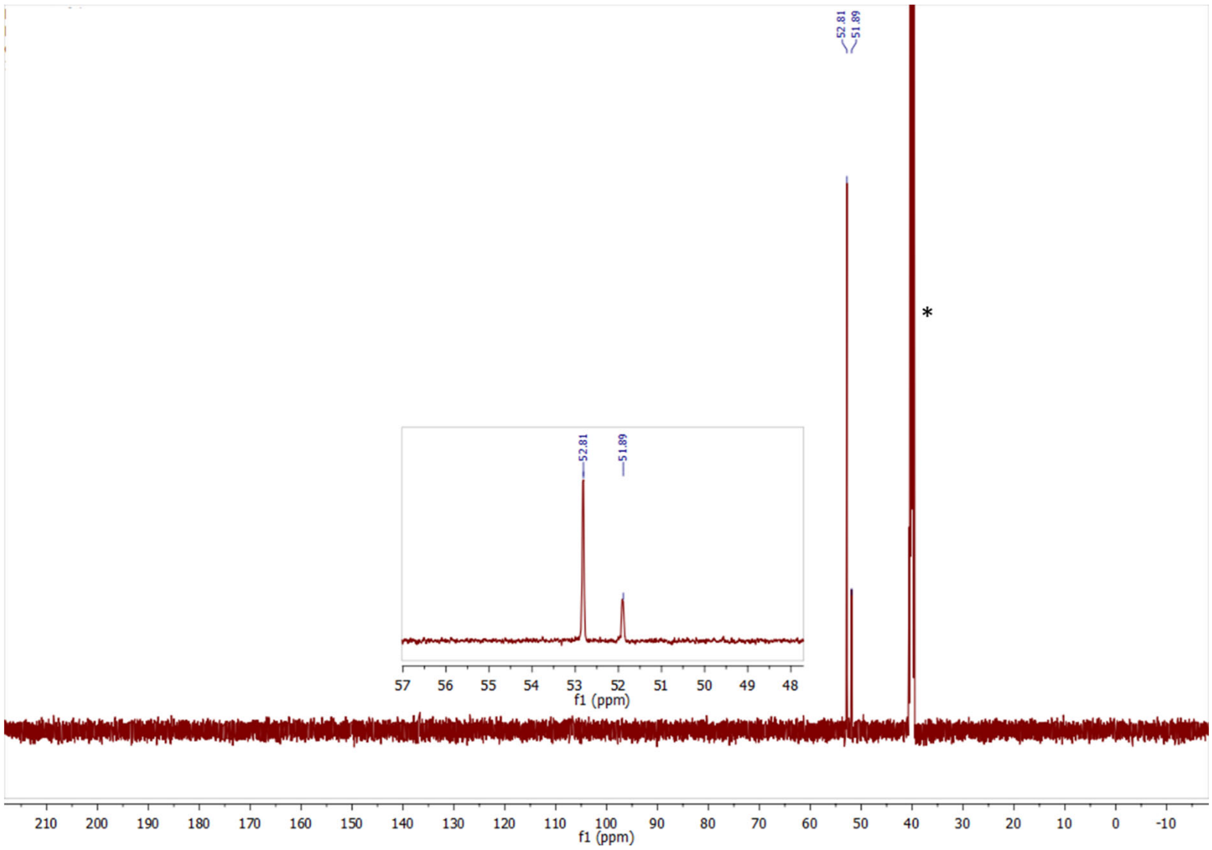

Picture 27: ^{13}C NMR spectrum of **5c** (* denotes residual solvent peak).

DBU dibromoiodate(I) (6c):

Dark orange sticky crystalline solid, yield 98 %.
^{1}H NMR (600 MHz, DMSO-d_6, 25 °C): δ 9.49 (s, 1H), 3.59 – 3.54 (m, 2H), 3.49 (t, *J* = 5.9 Hz, 2H), 3.26 (dq, *J* = 5.9, 3.2 Hz, 2H), 2.69 – 2.61 (m, 2H), 1.93 (p, *J* = 6.3 Hz, 2H), 1.74 – 1.57 (m, 6H).
^{13}C NMR (151 MHz, DMSO-d_6, 25 °C): δ 165.9, 53.9, 48.39, 38.1, 32.2, 31.18, 28.7, 26.4, 23.8, 19.4.
HRMS-ESI$^+$: 153.1386 $(M+H)^+$
Crystallization method: **6c** was dissolved in MeCN. The solution was placed in a capped bottle filled with Et_2O and into a refrigerator (4 °C). Crystals of **6c** formed in the solution within a week. However, when warmed to room temperature crystals melted/dissolved immediately.

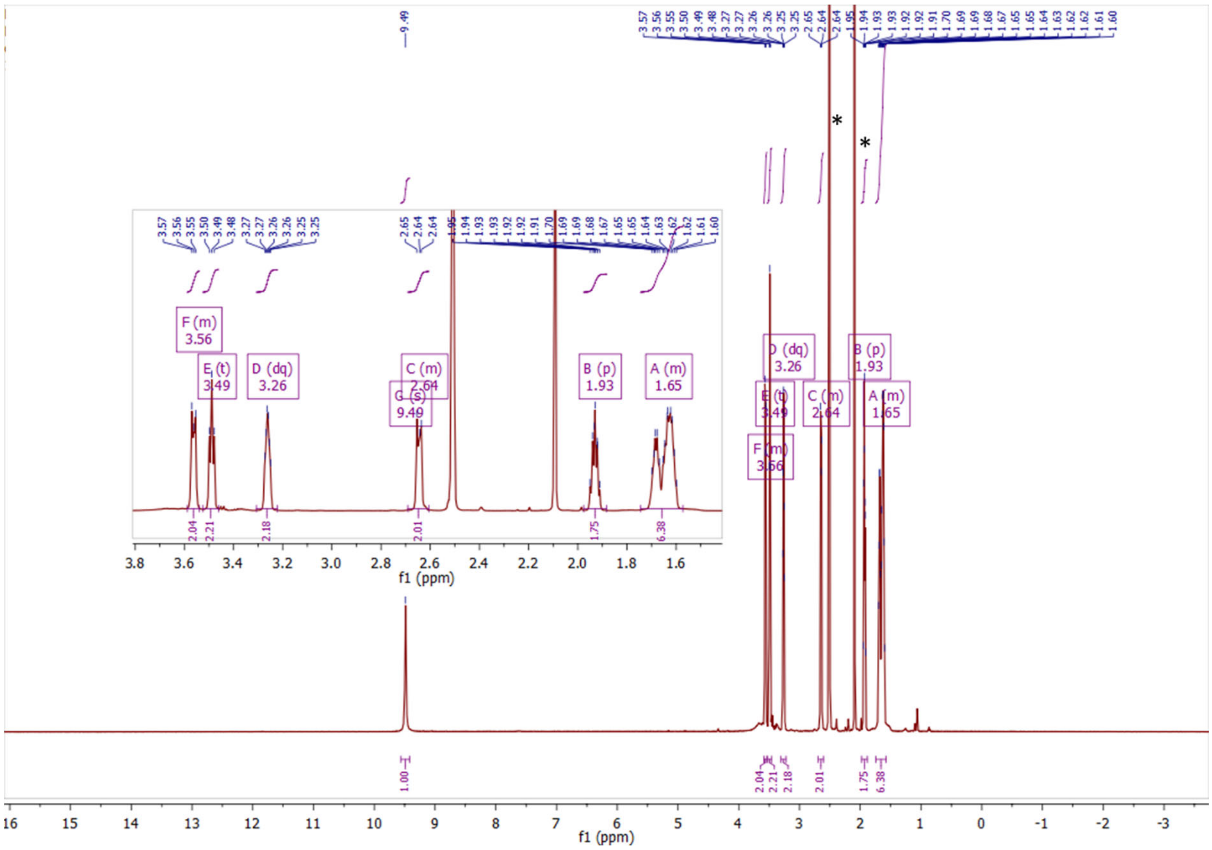

Picture 28: ^{1}H NMR spectrum of **6c** (* denotes residual solvent peaks).

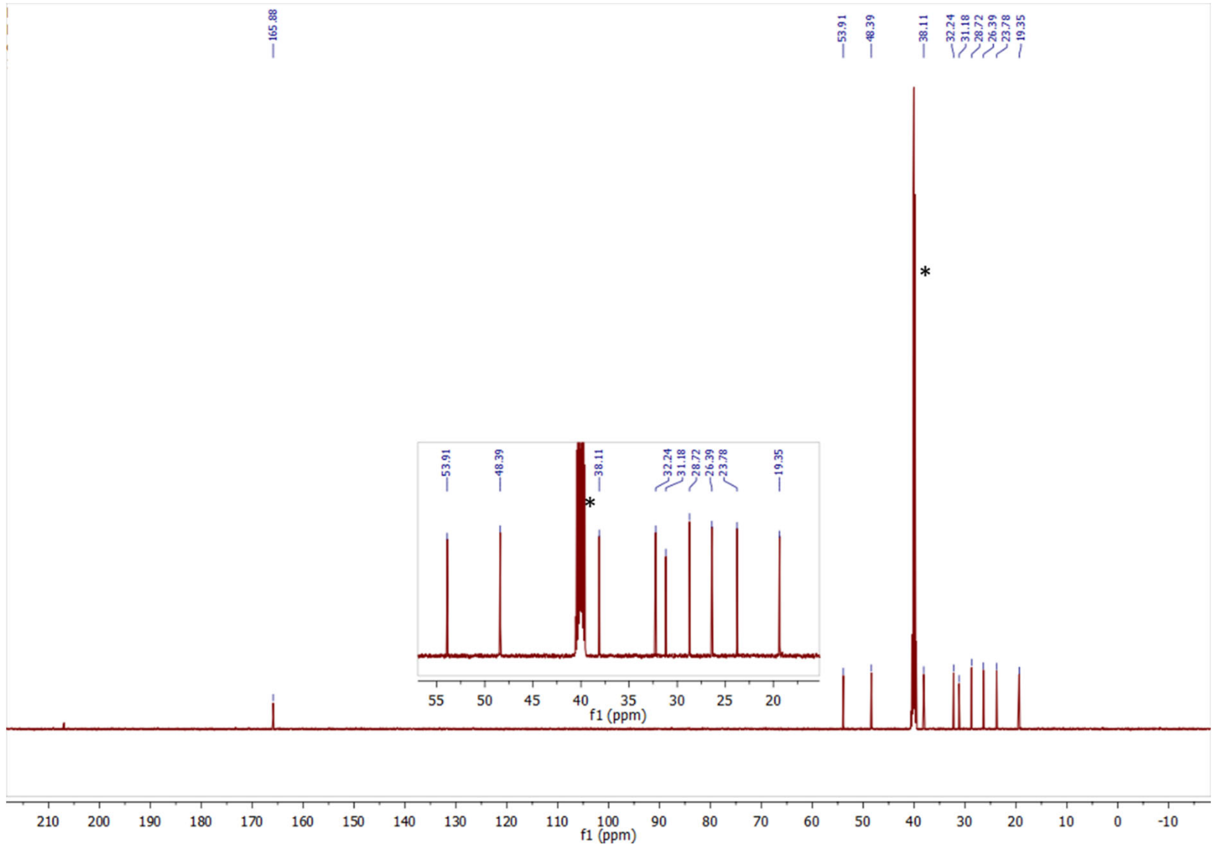

Picture 29: ^{13}C NMR spectrum of **6c** (* denotes residual solvent peaks).

Spectroscopic data for dichlorobromate(I) salts

2,4,6-tri-*tert*-butylpyridine dichlorobromate(I) (1d):

Light yellow powder, yield 50 %.
^{1}H NMR (600 MHz, DMSO-d_6, 25 °C): δ 11.72 (s, 1H), 7.68 (s, 2H), 1.68 (s, 18H), 1.46 (s, 9H).
^{13}C NMR (151 MHz, DMSO-d_6, 25 °C): δ 29.2, 30.2, 37.4, 119.3.
HRMS-ESI^{+}: 248.2373 (M+H)$^{+}$
Raman: 171 cm^{-1}.
Crystallization method: **1d** was dissolved in MeCN. The solution was placed in a capped bottle filled with Et_2O and into a refrigerator (4 °C). Crystals of **1d** formed in the solution within a week.

Crystallization method 2: **1d** was dissolved in DCM and the solvent was let to evaporate slowly at 4 °C in a refrigerator. Crystals of **1d** formed within a few days on the walls of the glass vial.

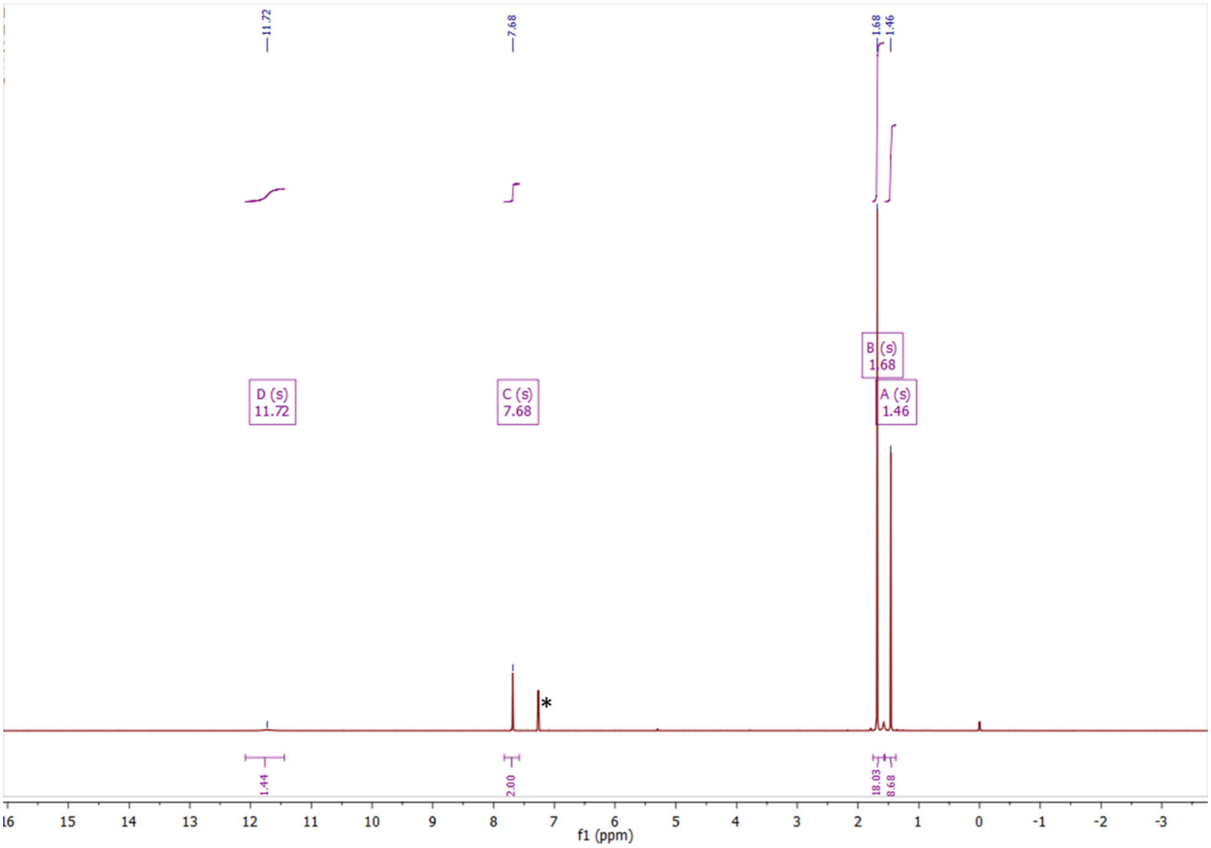

Picture 30: ^{1}H NMR spectrum of **1d** (* denotes residual solvent peaks).

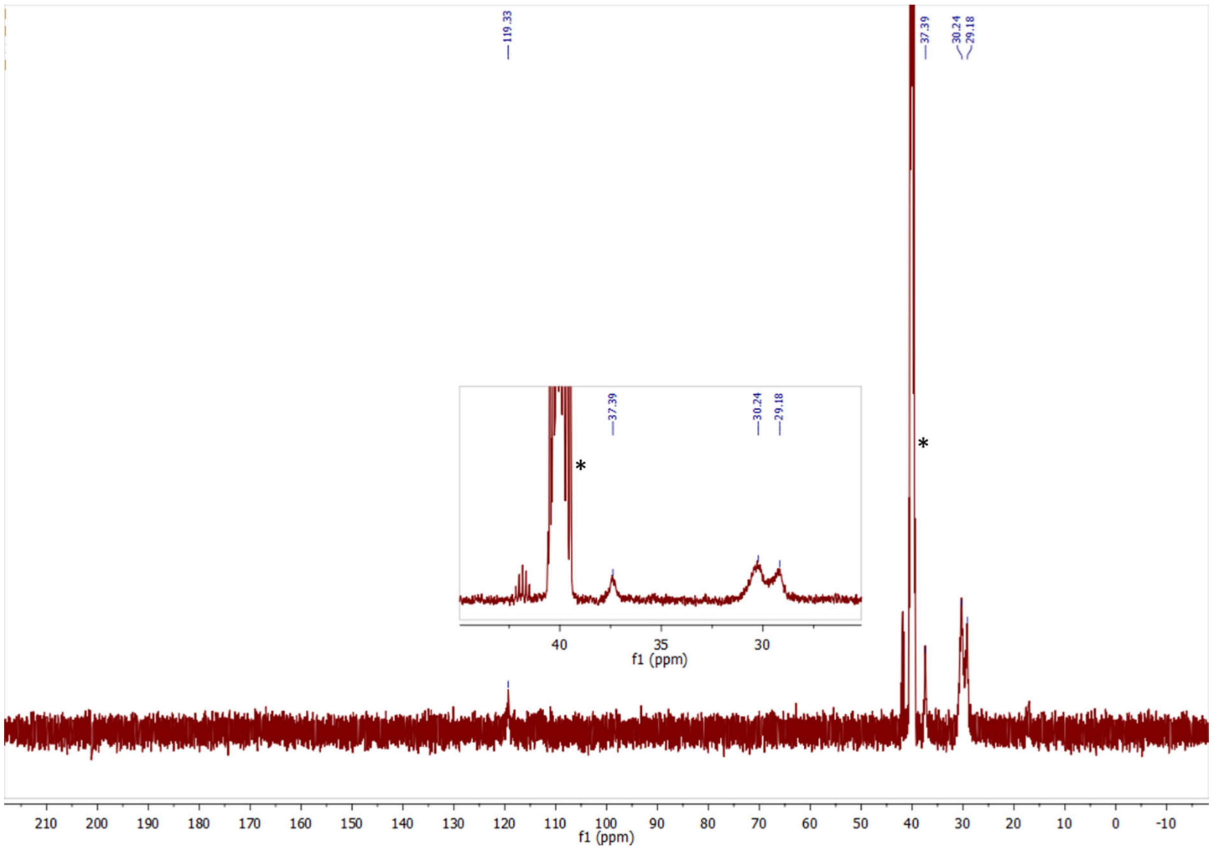

Picture 31: ^{13}C NMR spectrum of **1d** (* denotes residual solvent peaks).

References

[1] CrysAlisPro, Agilent Technologies, Version 1.171.37.31 (release 14-01-2014 CrysAlis171 .NET).

[2] Sheldrick, G.M., SHELXT - Integrated space-group and crystal-structure determination. *Acta Cryst*. **2015**, A71:3–8.

[3] Dolomanov OV, Bourhis LJ, Gildea RJ, Howard, JAK, Puschmann H, OLEX2: a complete structure solution, refinement and analysis program *J. Appl. Cryst.* (2009), 42*:*339–341.

[4] Sheldrick GM, Crystal structure refinement with SHELXL, *Acta Crystalogr. Sec. C* (2015) 71*:*3−8.

[5] Diamond - Crystal and Molecular Structure Visualization Crystal Impact - Dr. H. Putz & Dr. K. Brandenburg GbR, Kreuzherrenstr. 102, 53227 Bonn, Germany http://www.crystalimpact.com/diamond.

[6] Spek AL, Single-crystal structure validation with the program PLATON. J. Appl. Cryst. (2003) 36:7−11.

[7] Sreeperumbuduru RS, Abid ZM, Claunch KM, Chen HH, McGillivray SM, Simanek EE, *RSC Advances* (2016) 6:8806−8810.

[8] Ali Ghumro S, Alharthy RD, al-Rashida M, Ahmed S, Malik MI, Hameed A, *ACS Omega* (2017) 2*:* 2891−2900.
